# Unveiling yellow rust resistance in the near-Himalayan region: insights from a nested association mapping study

**DOI:** 10.1007/s00122-025-04886-z

**Published:** 2025-06-05

**Authors:** Katharina Jung, Reiko Akiyama, Jilu Nie, Miyuki Nitta, Naoto-Benjamin Hamaya, Naeela Qureshi, Sridhar Bhavani, Thomas Wicker, Beat Keller, Masahiro Kishii, Shuhei Nasuda, Kentaro K. Shimizu

**Affiliations:** 1 Department of Evolutionary Biology and Environmental Studies, University of Zurich, Zurich, Switzerland; 2 Graduate School of Agriculture, Kyoto University, Kyoto, Japan; 3 CIMMYT, El Batán, Mexico; 4 CIMMYT, Nairobi, Kenya; 5 Department of Plant and Microbial Biology, University of Zurich, Zurich, Switzerland; 6 Japan International Research Center for Agricultural Sciences (JIRCAS), Tsukuba, Japan; 7 Kihara Institute for Biological Research, Yokohama City University, Yokohama, Japan

## Abstract

**Key message:**

This study identified two potentially novel yellow rust resistance loci in traditional Asian wheat varieties and gives insights into the distribution of resistances in high disease-pressure regions near the Himalayas.

**Abstract:**

The global spread of yellow rust has posed a significant threat to wheat production, making the identification of novel resistance-conferring genetic loci crucial. The near-Himalayan region has been proposed as the pathogen’s origin and is characterized by strong and diverse disease pressure. Even though this makes wheat varieties from this region likely to harbor resistance, Asian germplasm has been highly underrepresented in modern breeding. To explore this potential, we screened an Asian nested association mapping (NAM) population comprising traditional and modern wheat varieties under artificial epidemics in multiple field trials. Combined quantitative trait locus (QTL) mapping revealed the two resistance genes *Lr67/Yr46/Sr55* and *Lr34/Yr18/Sr57*, as well as two potentially novel yellow rust resistance loci. The resistant allele of the first one, located on chromosome 3D, is unique to a traditional variety from Nepal, while the second one, found on chromosome 5B, is present in several NAM families. The broad geographic distribution of this QTL across regions with high disease pressure suggests it may serve as a durable source of resistance. Strong observed resistances were conferred by a combination of several resistance loci, suggesting the stacking of resistances as a successful strategy in yellow rust hotspot areas.

**Supplementary Information:**

The online version contains supplementary material available at 10.1007/s00122-025-04886-z.

## Introduction

Bread wheat (*Triticum aestivum*) is an allohexaploid species, a product of two polyploidization events that contributed to its wide adaptability (Dubcovsky and Dvorak 2007; Akagi et al. 2022). However, despite its robustness, wheat remains susceptible to numerous pathogens and pests. Among these, the three rust diseases are considered as most destructive. While leaf rust (*Puccinia triticina* (*Pt*)) and stem rust (*Puccinia graminis* f. sp. *tritici* (*Pgt*)) are widespread, yellow rust (*Puccinia striiformis* f. sp. *tritici* (*Pst*)) causes the most significant damage, affecting around 88% of global wheat production (Beddow et al. 2015). The characteristic yellow stripes on wheat leaves, which give the pathogen its common name, yellow or stripe rust (YR), significantly reduce photosynthetic activity, impacting grain yield and quality (Bouvet et al. 2022). Over the past two decades, more aggressive and genetically diverse *Pst* races have emerged in numerous regions worldwide, leading to severe epidemics (Beddow et al. 2015; Ali et al. 2017).

One strategy to combat YR involves developing wheat varieties with genetic resistance. Over 80 YR resistance genes have been identified (McIntosh 2024). Most of these genes fall into the category of race-specific or all-stage resistance (ASR), which the pathogen often overcomes through the boom-and-bust cycle (Van der Plank 1963). In contrast, some resistance genes belong to the adult plant resistance (APR) category, with several demonstrating pleiotropic resistance against multiple diseases, making them invaluable resources for breeding (Huerta-Espino et al. 2020; Bhavani et al. 2022). There is a critical need to identify new and durable sources of resistance genes to stay ahead of the ever-evolving *Pst* races. This study aims to uncover novel potential resistance loci and to highlight a geographic region crucial for generating and conserving durable sources of YR resistance for the future.

Asian germplasm has not been utilized significantly in modern wheat breeding despite its great potential for advancement due to its genetic diversity (Balfourier et al. 2019; Sansaloni et al. 2020; Lehnert et al. 2022; Okada and Shimizu 2024). Wheat varieties from the southern Himalayan regions and the Chinese lowlands, in particular, hold promise due to their location at YR hot spots. These geographic areas have either been proposed as the pathogen’s origin or are characterized by very high and diverse disease pressure (Ali et al. 2014, 2017; Thach et al. 2016; Li et al. 2021a). The YR pathogen exhibits a complex population structure in this region, with no detectable admixture between populations from the Tibetan highlands and the Chinese lowlands. There is only limited gene flow between populations from Pakistan, Nepal, and China (Hu et al. 2017; Khan et al. 2019; Awais et al. 2023). While the pathogen’s global and regional distribution is well-documented, the distribution of resistance genes in wheat, the host plant, remains less understood. The high disease pressure in these regions may have shaped the genetic makeup of regionally adapted traditional varieties, potentially playing a crucial role in the co-existence of host and pathogen. A better idea of the geographic distribution of resistance genes could inform targeted conservation efforts, helping to safeguard resistance sources for future breeding endeavors.

Since the development and initial implementation of a nested association mapping (NAM) population in maize, several NAM populations have been generated across various crops, demonstrating this design’s effectiveness in identifying genetic loci associated with a wide range of traits (Yu et al. 2008; Buckler et al. 2009). The NAM design overcomes some limitations of traditional mapping approaches: First, NAM populations capture more genetic diversity of a species by incorporating multiple parental cultivars, unlike bi-parental mapping, which uses only two. Second, NAM mapping is based on experimentally induced recombination events during the construction of the population, like bi-parental quantitative trait locus (QTL) mapping, which contrasts with the correlational evidence of historical recombination used in genome-wide association studies (GWAS) (Gage et al. 2020). Therefore, NAM mapping combines the strengths of both bi-parental QTL mapping and GWAS, providing robust evidence of the mapped loci within each parental cultivar.

The Asian NAM population in wheat was developed by crossing the common parent Norin 61 (N61), an elite Japanese variety with a previously reported chromosome-scale genome assembly (Walkowiak et al. 2020; Tsujimoto 2021; Shimizu et al. 2021), with a diverse set of 24 traditional and modern varieties from Nepal, Pakistan, China, and Japan (Nie  2025; Nomura et al. 2025). This NAM population, which includes at least seven founder lines from the region where the YR pathogen likely originated, represents a unique resource for identifying new resistance loci within the near-Himalayan region. Additionally, it allows us to observe the distribution of individual loci within the population, an advantage not possible with a bi-parental mapping population.

Utilizing the Asian wheat NAM population, this study had two primary aims: first, to identify the genomic basis of rust resistance across different genetic backgrounds, and second, to uncover the distribution of rust resistance loci within our NAM population, including wheat lines originating from near-Himalayan regions with high disease pressure. Our study aimed to explore the following questions: Is YR resistance present in the NAM parents? Which genomic regions in the NAM population are linked to YR resistance, and have they been previously characterized? Do these genomic regions also provide resistance to leaf and stem rust? Which parental lines contributed to these loci in the NAM population? Can we identify distinct geographic patterns in the distribution of rust resistance loci?

## Material and methods

### Plant material

The common paternal line of the NAM population used in this study was the Japanese variety Norin 61 (Shimizu et al. 2021), while the maternal lines included 24 traditional and modern varieties from Pakistan, Nepal, Japan, and China (Nie 2025; Nomura et al. 2025; Table 1). The 25 varieties were chosen to broadly represent the diversity of the Asian core collection from the NBRP-Wheat genebank, based on the phylogeny published in Takenaka et al. (2018).

**Table 1:** Summary information about 25 parental lines of the Asian NAM population

Genotype	Abbreviation	Origin	Variety type	Pedigree	Vernalization
Norin 10*	N10	Japan	Modern	Turkeyred (USA) × Fultz Daruma	Yes
KU-479*	CN4	China	Traditional	–	No
Fukoku*	FKK	Japan	Traditional	–	Yes
KU-3299*	PK1	Pakistan	Traditional	–	No
KU-3351	PK2	Pakistan	Traditional	–	Yes
KU-4734*	NP1	Nepal	Traditional	–	Yes
KU-4769*	NP2	Nepal	Traditional	–	Yes
KU-4783	NP3	Nepal	Traditional	–	No
KU-7113*	NP4	Nepal	Traditional	–	No
KU-13546	CN2	China	Traditional	–	Yes
KU-13662*	CN1	China	Traditional	–	Yes
KU-13708	CN6	China	Traditional	–	Yes
KU-13807	CN7	China	Traditional	–	Yes
KU-13891	CN3	China	Traditional	–	Yes
Chinese Spring*	CS	China	Traditional	–	Yes
Kanto 107	K107	Japan	Modern	Kanto 79 × Kanto 82	No
Zenkojikomugi*	ZNK	Japan	Modern	Iga Chikugo × Oregon (USA) followed by gamma-ray radiation	No
Nobeokabouzu*	NBB	Japan	Traditional	–	Yes
Minaminokomugi	MNM	Japan	Modern	Seikai 113 × Fujimikomugi (= Kanto 56)	No
Chogokuwase	CGW	Japan	Modern	Geurumil × Minaminokomugi	No
Chikugoizumi	CKG	Japan	Modern	Asakazekomugi × Kanto 107	No
Shiroganekomugi*	SRG	Japan	Modern	Shirasagikomugi × Seikai 104	No
Shunyou*	SNY	Japan	Modern	Tohoku 148 × Tousan 10	Yes
Akadaruma	AKD	Japan	Traditional	–	Yes
Norin 61**	N61	Japan	Modern	Fukuokakomugi 18 × Shinchunaga	No

*Maternal lines of focal families

**Shared paternal line of NAM population

Adopting parts of the definition by Villa et al. (2005), we consider most NAM parents as traditional varieties, also referred to as “landraces”, which have been collected from traditional farming systems and did not undergo systematic breeding. All NAM parents from China, Pakistan, and Nepal match this definition. Most Japanese NAM parents were developed after the beginning of wheat breeding in Japan at the beginning of the twentieth century and are therefore considered modern varieties here (Fukunaga and Inagaki 1985, Supplementary Fig. 1). However, the two Japanese varieties, AKD and FKK, were developed during the early stages of wheat breeding in Japan (1901 and 1912, respectively) and were likely derived from domestic varieties through pure line selection prior to the first systematic crosses in the 1910s (Zeven and Zeven-Hussink 1976, Noda 1986; Kojima et al. 2017). Therefore, they are considered traditional varieties here. NBB was previously reported as a landrace and is therefore also categorized as a traditional variety in this study (NARO 2024).

All maternal NAM parents described above were crossed to Norin 61 to develop the NAM population, which was followed by seven to eight cycles of self-propagation and resulted in 24 families, each consisting of 50 to 160 individual recombinant inbred lines (RILs). For this study, a subset of 13 families comprising 1060 lines was selected, representing the genetic and geographic diversity of the NAM population (Supplementary Table 1).

From these 13 NAM families, four originated from genotypes that were collected at the proposed origin of the yellow rust pathogen, i.e., south of the Himalayan mountains (PK1, NP1, NP2 and NP4). Three NAM families originated from Chinese genotypes. Two of them were collected in the lowlands in the Sichuan region (CS and CN4), which is known for high yellow rust disease pressure. The third one was collected on the Tibetan Plateau in high altitudes (CN1), representing a geographically distinct accession from those on the southern side. Furthermore, six NAM families originated from Japanese genotypes that represent different geographic areas of Japan (N10, FKK, ZNK, NBB, SRG, and SNY).

### Field trials

To evaluate the adult plant response to YR infection in the NAM population and their parental lines, field trials were conducted in Mexico (Toluca) and Switzerland (Zurich) (Supplementary Table 2 and 3). The 25 NAM parents were screened over two years in Toluca (2020, 2021) and Zurich (2022, 2023), while the focal NAM lines underwent similar assessments in Toluca (2021, 2022) and Zurich (2022, 2023, Supplementary Table 3). In Toluca, the parental lines and focal NAM lines were tested separately, whereas in Zurich, the trials were combined. Genotypes were placed randomly in all unreplicated trials, and in replicated trials, they were laid out in a completely randomized block design.

To assess whether the identified genetic loci confer pleiotropic resistance to various rust pathogens, the NAM lines were tested under simultaneous leaf rust (LR) and stem rust (SR) infection in Ciudad Obregón, Mexico (2022) and under LR infection in El-Batan in Mexico (2022). During all field trials, the test entries were surrounded by a mixture of highly susceptible spreader varieties, and spatially repeated check varieties with known infection responses were included to ensure uniform disease pressure. All plots were artificially inoculated with the pathogen of interest using the rust types specified in Supplementary Table 3.

While lines requiring vernalization were excluded during the development of the NAM population, some of the NAM parental lines did require vernalization (Table 1). These parental lines received a six- to eight-week cold treatment (6–8 °C) before being transplanted to the field in Toluca. All other trials were directly sown in the field. Nitrogen, phosphorus and potassium fertilizers were applied following local standards, and no fungicides were applied.

In the YR and LR trials, disease severity was assessed on the flag leaves after heading once the spreader rows displayed pronounced disease symptoms. Severity was quantified as the percentage of infected leaf area (0–100%) using the modified Cobb scale, with the average severity recorded at the plot level (Peterson et al. 1948). In the SR trial, disease severity was assessed by scoring the percentage of symptomatic stem area (0–100%) once initial symptoms appeared. LR and SR infections were evaluated separately within the same trial. Each plot was scored multiple times, with the average score recorded at each time point. In trials with replicates, mean scores were calculated. The maximum disease severity score observed for each line was used for subsequent analysis.

### Seedling trials

The NAM parents were grown in a greenhouse and artificially inoculated after the seedlings reached two leaf stage with the two predominant yellow rust races MEX14.191 and MEX16.04 in separate trials. 14 days after infection their resistance response was evaluated on a scale from 0 to 4 and the appearance of chlorosis was reported, following McIntosh et al. (1995).

### Analysis of phenotypic data

Data analyses were conducted using the R Statistical Software version 4.3.2 (R Core Team 2023). Mixed models were performed utilizing the “lme4” package (Bates et al. 2015). The year and location combinations were considered distinct environments, and disease infection scores were converted into fractions. An analysis of variance (ANOVA) was performed on the replicated parental lines to identify factors contributing to variation in disease severity, with the disease severity score as the response variable. The mixed model treated genotype, environment, and genotype-by-environment interaction as fixed effects, while replicates were considered random. Spearman’s correlation of disease severity scores between environments was calculated separately for both NAM parents and NAM lines to evaluate the stability of the lines across different environments.

The broad-sense heritability (*H*^*2*^) of the disease severity score was estimated within the replicated trials of the parental lines. Variance components were extracted from a mixed model in which genotype, environment, and genotype-by-environment interaction were treated as random effects. *H*^*2*^ was estimated using the formula from Hallauer et al. (2010), with ${\sigma }_{g}^{2}$, ${\sigma }_{e}^{2},$ and ${\sigma }_{ge}^{2}$ representing the phenotypic variance of genotype, environment, and genotype-by-environment interaction respectively, $r$ as the number of replicates, and $e$ the number of environments:

$$H^{2} = \frac{{\sigma_{g}^{2} }}{{\frac{{\sigma_{e}^{2} }}{re} + \frac{{\sigma_{ge}^{2} }}{e} + \sigma_{g}^{2} }}$$

### Consensus map

A detailed description of the consensus map construction can be found in Nie (2025) but a brief overview is provided here: Leaf samples from 1060 NAM lines at the F_7_ generation were collected at the two-leaf stage. DNA was extracted from single plants of pure lines that have been conserved by repeated selfing in the NBRP-Wheat genebank using the DNeasy Plant Mini Kit (Qiagen) and genotyped with GRAS-Di (Enoki and Takeuchi 2018; Hosoya et al. 2019). The fastp software (v0.20.0 (Chen 2023)) was used to detect and remove adapters from the raw reads. Subsequently, the *T. aestivum* ‘Norin 61’ reference genome (GCA_904066035.1) was employed as the reference, and alignment and sorting were performed using BWA-mem (v0.7.17 (Li and Durbin 2009)) and SAMtools (v1.1 (Danecek et al. 2021)). SNP calling was carried out using GATK (v4.2 (Poplin et al. 2018)). Individual linkage maps were constructed using IciMapping (v4.2 (Meng et al. 2015)). Finally, single maps were merged into a consensus map using the R package LPmerge (Endelman and Plomion 2014).

### QTL mapping

QTL mapping was done using the R package “statgenMPP,” designed explicitly for multi-parent populations. It utilizes a mixed model approach based on identity-by-descent (IBD) calculations while accounting for family kinship (Li et al. 2021b, 2022). To compute IBD blocks in 1 cM increments, inputs included the consensus map, genotype information of all lines, the population pedigree, and the propagation generation. Using default settings, a chromosome-specific kinship matrix was calculated based on the IBD probabilities. This matrix was adjusted for each trait by excluding lines with missing phenotype data.

To establish a significance threshold for QTL mapping, a 1000 permutation test was conducted, with phenotypes randomly shuffled independently of genotypes in each iteration. The highest LOD score from each run was recorded, and the 95th percentile of these scores was set as the significance threshold for the trait. Due to its computational intensity and minimal impact, the kinship matrix was excluded from the single QTL mapping during the permutation test (Supplementary Fig. 2).

Finally, QTL mapping for multiple loci of each trait was performed using the IBD probabilities and the kinship matrix. These analyses were conducted separately for the full set of lines (1060 lines) and for the Zurich subsets from 2022 (350 lines) and 2023 (710 lines). QTL intervals are defined as the full width of each significant peak, from the sharp LOD score rise at the start to the peak’s end. The family-wise QTL effects are based on the hidden ancestral allele, or haplotype, at each locus, also known as the “latent ancestral origin state”. These effects are estimated across all families using a Hidden-Markov model framework (Zheng et al. 2015).

### Identifying physical positions of QTLs

The closest flanking markers on both sides of the QTL region on the consensus map were identified to determine the physical position of the detected QTLs. All markers within this interval, including those at the same linkage position as the flanking markers, were extracted. Given that the physical position of each marker on Norin 61 is known, the QTL’s physical location could be defined using the extracted markers. If the marker’s physical position deviated significantly from surrounding markers, potentially due to rearrangements, it was excluded from consideration.

### DNA extraction and marker-based genotyping

Wheat leaf tissue from the NAM parents was collected from plants grown in a greenhouse at the University of Zurich, Switzerland and then stored at − 20 °C until processing. The leaves were frozen in liquid nitrogen and pulverized with glass beads before DNA extraction using the DNeasy Plant Mini kit by Qiagen as per instructions.

Genotyping for *Lr34/Yr18/Sr57* was carried out using the molecular markers *cssfr1* and *cssfr2* that target a 3-base pair deletion within the exon 11 (Lagudah et al. 2009), as well as the markers *caSNP4* and *caSNP12* that identify SNPs within intron 4 and exon 12 (Dakouri et al. 2010). For *Lr67/Yr46/Sr55* genotyping, the marker *cfd71*, described by Hiebert et al. (2010), was used. The marker *InDel_SRPK* from Long et al. (2024) was employed to genotype the previously named gene *YrAYH* on chromosome 5B. GoTaq polymerase was used for amplification; detailed conditions are in Supplementary Tables 4 and 5. Results for *cfd71* were visualized using MultiNA (MCE-202, Shimadzu, Japan) with a DNA-500 reagent kit, while all other markers were resolved on an agarose gel.

### Comparison of physical positions with previously published genes and QTLs

Information about the physical position or the position of flanking markers on the Chinese Spring reference genome (IWGSC RefSeq v1.0) was gathered from the literature for previously published QTLs. A region spanning 1,000 bp at the beginning and the end of our QTL regions was extracted from the Norin 61 reference genome and aligned to the Chinese Spring reference genome using BLAST to determine the physical positions on Chinese Spring. The same was conducted vice versa to identify the physical position of *Lr34/Yr18/Sr57* and *Lr67/Yr46/Sr55* on the Norin 61 reference genome.

## Results

### YR resistance present in NAM parents from areas of high disease pressure

**Fig. 1:**
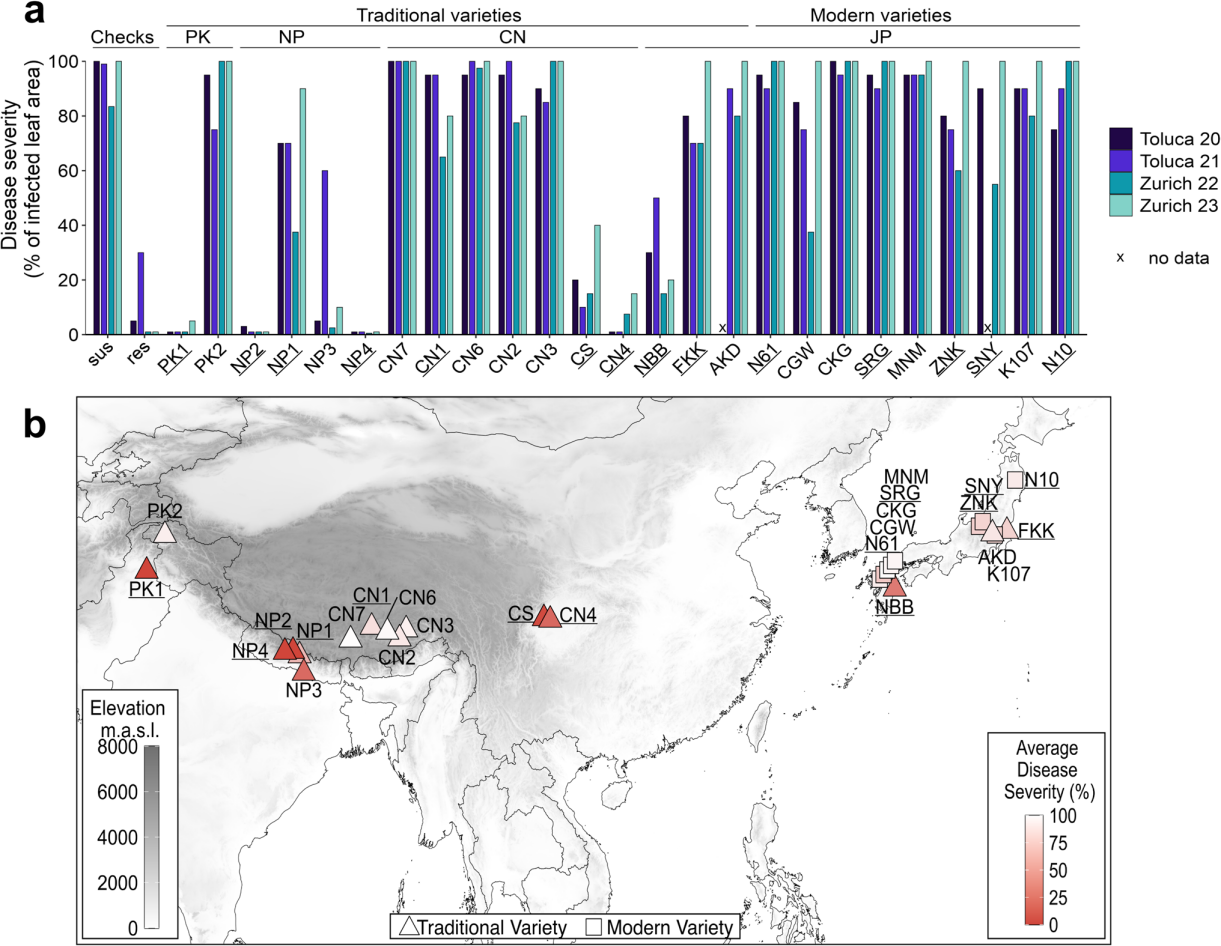
**a** Disease severity (% of infected leaf area) under artificial YR infection was assessed in NAM parents across two locations, Toluca and Zurich, over two years. In Toluca, Mugami (sus) and Quaiu (res) served as checks, while in Zurich, Eridano (sus) and Nara (res) were used. Labels above the plot indicate the origin of genotypes (JP = Japan, CN = China, PK = Pakistan, NP = Nepal) and whether they are categorized as modern or traditional varieties. **b** Disease severity (% of infected leaf area) of NAM parents under artificial YR infection averaged over the four environments is indicated by the color gradient and plotted at the geographic origin of each NAM parent. A more robust red indicates a highly resistant genotype, while the white ones are entirely susceptible. The shapes define if the genotype is considered a modern or traditional variety. The gray color gradient represents the elevation. In both plots, the genotypes selected for further trials are underlined

The 25 parental genotypes of the Asian NAM population were assessed for disease severity (% infected leaf area) under controlled artificial epidemics at two locations over two years (Supplementary Table 6). A wide range of responses to yellow rust (YR) infection was observed among these genotypes (Fig. 1a). All modern varieties from Japan, including the common parental line Norin 61, as well as two traditional Japanese varieties (AKD and FKK), all Chinese varieties from the Tibetan highlands (CN1, CN2, CN3, CN6, CN7), and one variety from the high mountains of Pakistan (PK2), were found susceptible or exhibited only partial resistance (Fig. 1b).

Conversely, three traditional varieties from south of the Himalayan mountain ranges (PK1, NP2, NP4) displayed near-immunity. Intermediate quantitative resistance was observed in a traditional variety from Japan (NBB), two varieties from the Chinese lowlands (CS and CN4), and two varieties from Nepal (NP1 and NP3).

Susceptible checks were heavily infected across all environments, confirming the efficacy of artificial epidemics. Resistant checks consistently demonstrated strong resistance in all environments.

No striking difference was visible between experimental locations and years, except for some heavier infections in the second year in Zurich in some Japanese varieties and the traditional varieties CS and NP1. YR disease resistance for all genotypes was highly correlated between all environments, i.e., location by year combinations (Supplementary Table 7).

The analysis of variance (ANOVA) demonstrated significant effects of genotypes, environments, and their interaction on YR resistance (Supplementary Table 8). Genotype was identified as the primary source of variation in disease response. The broad-sense heritability (*H*^*2*^) of the maximum disease score among the NAM parents across all environments was notably high, estimated at 0.96.

### Segregation in NAM lines for yellow rust, leaf rust, and stem rust

Thirteen NAM families, representing the genetic and geographic diversity of the NAM population, were selected as the focal families and evaluated for their response to YR, leaf rust (LR), and stem rust (SR) at the adult plant stage.

**Fig. 2:**
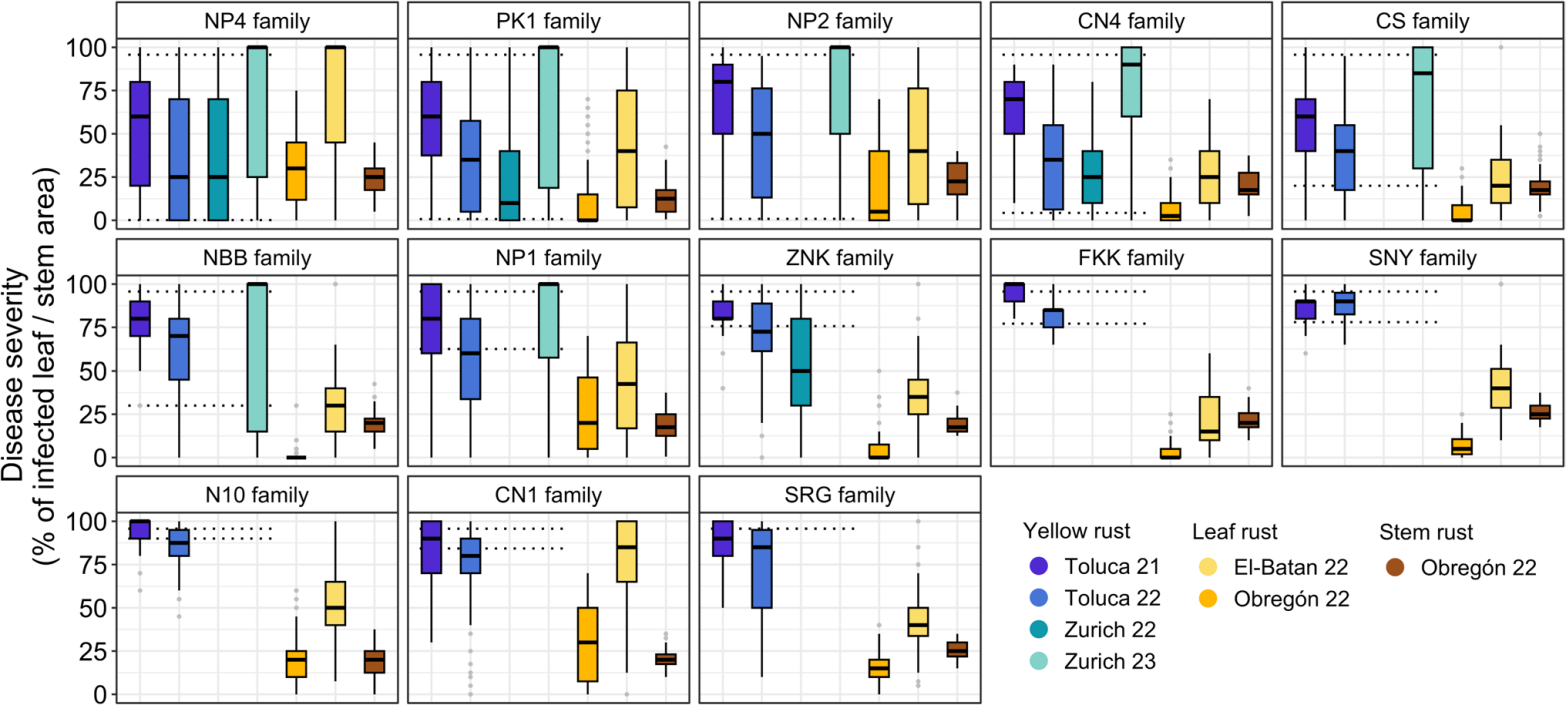
Disease severity (% of infected leaf/stem area) scored in NAM families for YR, LR, and SR over three years. Environments are visualized in different colors. The dotted lines represent the mean disease severity of the NAM parents for YR: the line that is the same in all plots represents Norin 61. In contrast, the line that varies between families represents the corresponding alternative parent

Field trials assessing YR resistance in the NAM lines were conducted for two years at Toluca, Mexico, and Zurich, Switzerland (Supplementary Table 9). These trials indicated stronger YR severity in Toluca 21 and Zurich 23, as evidenced by higher mean disease severity for all families (Fig. 2). Some families displayed a broad spectrum of resistance, from completely susceptible to fully resistant. Families with various levels of partial resistance were identified, with resistance broadly correlating with the mean range defined by their parent lines (CS, NBB, NP1, and ZNK). Conversely, families such as the FKK family, the SNY family, the N10 family, and the CN1 family appeared to lack significant YR resistance genes.

The LR data showed a markedly stronger LR disease severity in El-Batan 22, reaching a maximum of 100% infected leaf area, compared to a maximum of about 75% in Obregón 22 (Fig. 2). This could be attributed to SR infections overlapping with LR infections, resulting in a higher disease response. Japanese parent-derived families (NBB, ZNK, FKK, SNY, N10, SRG) and those from the Sichuan region in China (CN4, CS) exhibited significantly lower LR infection than other families.

For SR infections, levels were relatively low across all families, with a mean of approximately 25% infected stems. The PK1 family, originating from a traditional Pakistani variety, was a notable exception, showing considerably lower infection levels (Fig. 2).

High correlations within the YR and LR scores of NAM lines in different environments suggest the presence of some relatively stable resistance loci in the population (Table 2). Overall, correlations within individual families for YR were high, except for three highly susceptible families (FKK, SNY, N10) with low phenotypic variation (Supplementary Table 10). Most families exhibited intermediate to high within-family correlations for LR, except those originating from NBB, ZNK, N10, and SRG (Supplementary Table 11). The low correlations for these four families could be attributed to the reduced phenotypic variation recorded in Obregón 22, potentially due to the cross-infection with LR and SR (Fig. 2).

**Table 2:** Spearman’s correlation for YR disease severity score (right) of NAM lines over four environments (a) and LR disease severity score (left) of NAM lines over two environments (b)

(a)	Toluca 22	Zurich 22	Zurich 23	(b)	Obregón 22
Toluca 21	0.78***	0.70***	0.53***	El-Batan 22	0.66***
Toluca 22		0.72***	0.62***		
Zurich 22			0.57***		

***P ≤ 0.001

### Multiple QTLs detected for all rust diseases

**Fig. 3:**
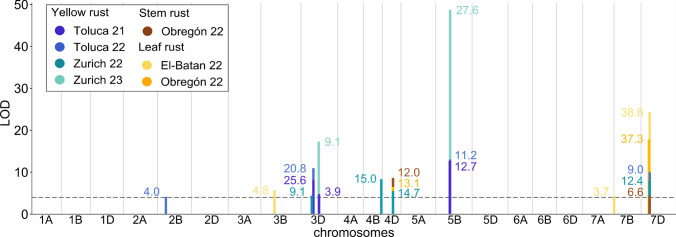
Significant QTL peaks from consensus mapping for YR, LR, and SR infection scores (% of infected leaf/stem area) collected in seven environments, which are represented by color. The horizontal dashed line indicates the mean significance threshold over all locations (based on permutation tests), and the numbers close to the peaks indicate the amount of phenotypic variation (PVE %) explained by the corresponding QTLs

Combined QTL mapping was conducted for all families using the maximum disease severity scores for YR, LR, and SR in each environment. Nine significant loci were identified, some of them consistent across different years and locations (Fig. 3, Table 3, Supplementary Table 12). Full mapping results for each environment can be found in Supplementary Fig. 3 and a detailed representation of each significant peak in Supplementary Figs. 4 to 12.

**Table 3:** QTLs detected by consensus mapping in the NAM population for field resistance to yellow rust (Toluca 21, Toluca 22, Zurich 22, Zurich 23), leaf rust (El-Batan 22, Obregón 22), and stem rust (Obregón 22) at adult plant stage

	Pathogen	Location	Position (cM)	Peak (cM)	Position (Mb)	LOD	PVE (%)
*QYr.uzh-2B*	Yellow rust	Toluca 22	42.5–57.6	50.0	32.05–53.66	4.1	4.0
*QLr.uzh-3B*	Leaf rust	El-Batan 22	73.8–86.5	79.9	462.04–651.84	5.7	4.8
*QYr.uzh-3D.1*	Yellow rust	Toluca 21	61.0–75.2	68.7	56.15–331.54	8.0	25.6
	Yellow rust	Toluca 22	66.4–79.2	72.7	76.66–359.27	11.0	20.8
	Yellow rust	Zurich 22	55.2–69.6	62.7	47.53–135.07	4.4	9.1
*QYr.uzh-3D.2*	Yellow rust	Toluca 21	99.5–112.7	105.6	459.88–533.73	4.7	3.9
	Yellow rust	Zurich 23	97.5–109.8	103.6	457.38–533.73	17.3	9.1
*QYr.uzh-4B*	Yellow rust	Zurich 22	95.4–110.0	104.1	651.08–669.73	8.1	15.0
*QLrYrSr.uzh-4D*	Yellow rust	Zurich 22	56.5–70.3	62.6	342.08–450.72	5.5	14.7
	Leaf rust	Obregón 22	57.6–70.3	63.6	351.51–450.72	6.5	13.1
	Stem rust	Obregón 22	57.6–70.3	63.6	351.51–450.72	8.6	12.0
*QYr.uzh-5B*	Yellow rust	Toluca 21	76.5–90.1	82.8	514.58–538.59	12.7	12.7
	Yellow rust	Toluca 22	79.8–91.9	85.8	523.26–543.91	13.0	11.2
	Yellow rust	Zurich 23	78.7–90.9	84.8	517.58–542.17	48.7	27.6
*QLr.uzh-7B*	Leaf rust	El-Batan 22	0.0–7.1	1.0	1.69–2.55	4.3	3.7
*QLrYrSr.uzh-7D*	Yellow rust	Toluca 22	43.2–62.0	51.9	53.55–82.84	10.0	9.0
	Yellow rust	Zurich 22	43.2–57.5	49.9	53.55–80.31	8.0	12.4
	Leaf rust	El-Batan 22	43.2–62.0	51.9	53.55–82.84	24.4	38.8
	Leaf rust	Obregón 22	38.3–54.1	46.9	39.68–80.31	17.8	37.3
	Stem rust	Obregón 22	43.2–57.5	49.9	53.55–80.31	4.4	6.6

The physical position is located on the Norin 61 reference genome

PVE (%) represents the phenotypic variation explained by each QTL

Two pleiotropic loci were detected, conferring resistance to all three rust diseases (Fig. 3). *QLrYrSr.uzh-7D* was identified on the long arm of chromosome 7D, co-located in different environments. Another locus was found on the long arm of chromosome 4D (*QLrYrSr.uzh-4D*), overlapping in different environments and explaining almost the same amount of phenotypic variation (PVE) for all three diseases.

A QTL specifically conferring resistance only to YR was identified on the long arm of chromosome 5B (*QYr.uzh-5B*), with the highest LOD score of around 50 in Zurich 23. Significant QTLs overlapping the same genomic region were also identified in Toluca 21 and Toluca 22, although the LOD scores were lower in these environments. The prominent peak in Zurich 23 explains approximately 28% of PVE, while the peaks in Toluca are responsible for much lower PVE (~ 12%).

On chromosome 3D, two QTLs were identified, both reducing the YR severity. They will hereafter be referred to as *QYr.uzh-3D.1* (in the pericentromeric region of the short arm) and *QYr.uzh-3D.2* (on the long arm). *QYr.uzh-3D.1* was significant in Toluca and one year in Zurich, indicating a relatively consistent QTL across environments. However, the explained PVE in Toluca (20.8 to 25.6%) was much higher than in Zurich (9.1%). *QYr.uzh-3D.2* also appeared stable in both locations but explained much less PVE than *QYr.uzh.3D.1*.

Four additional minor QTLs were identified for YR and LR, each significant in single environments. For YR, QTLs were detected on chromosomes 2B and 4B, while QTLs on chromosomes 3B and 7B were detected in LR trials. Except for the QTL on chromosome 4B, they all explained a very low PVE of less than 5%. In the following analysis, the focus will be on the four major loci, which are *QYr.uzh-3D.1*, *QLrYrSr.uzh-4D*, *QYr.uzh-5B* and *QLrYrSr.uzh-7D*.

### Physical positions of main QTLs on Norin 61

Assessing the physical positions of the four main QTLs revealed that two overlap with previously cloned resistance genes (*QLrYrSr.uzh-7D* and *QLrYrSr.uzh-4D*), while two are possibly novel loci (*QYr.uzh-3D.1* and *QYr.uzh-5B*).

**Fig. 4:**
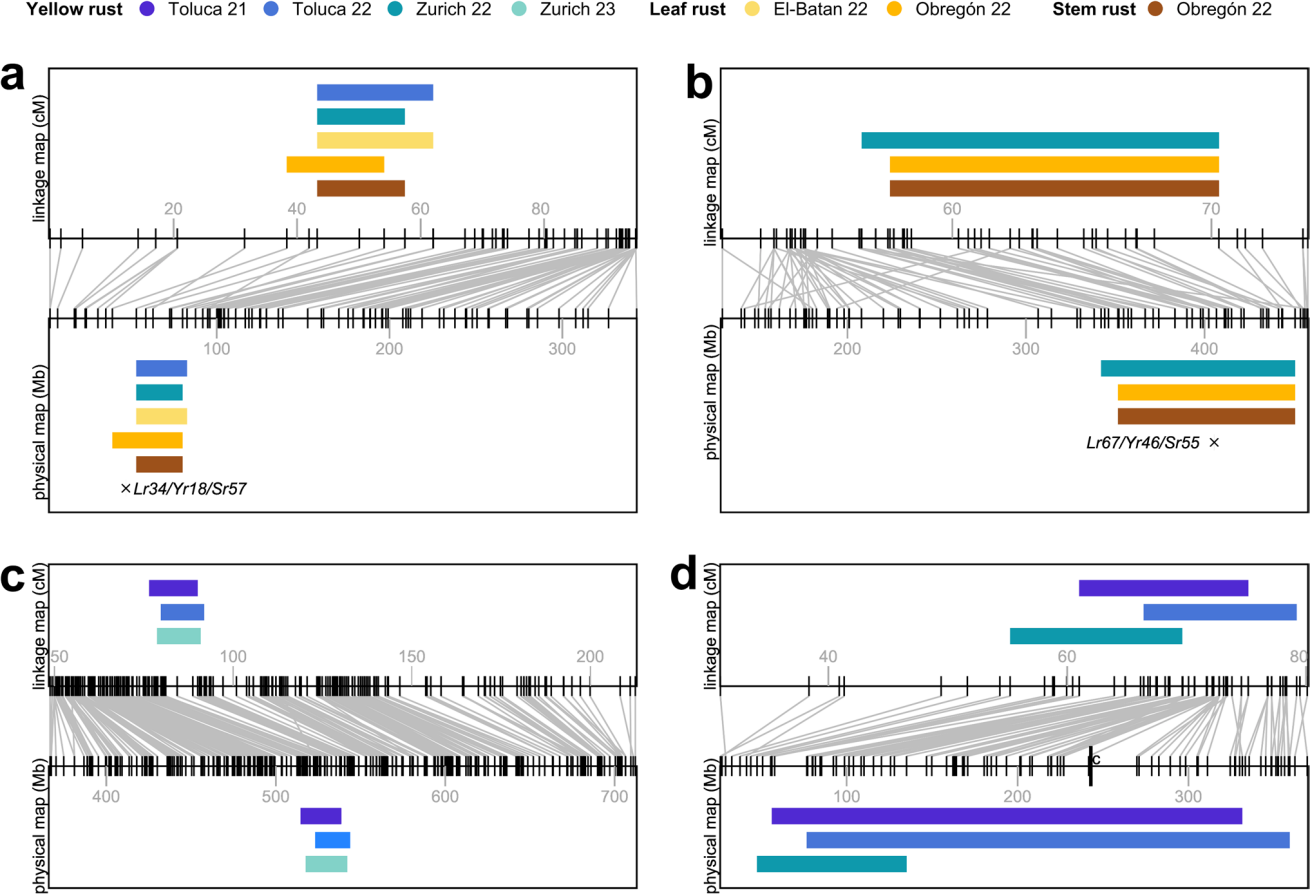
Positions of **a** *QLrYrSr.uzh-7D*, **b** *QLrYrSr.uzh-4D*, **c** *QYr.uzh-5B*, and **d** *QYr.uzh-3D.1* are shown as colored bars on the consensus map (centimorgans (cM), upper box) and the physical map of the common parent Norin 61 (megabases (Mb), lower box). QTL intervals are defined as the full width of each significant peak, from the sharp LOD score rise at the start to the peak’s end. The colors of QTLs correspond to the environments in which they were identified. Vertical black lines on both maps present observed markers, while the long vertical black line (marked with c in panel d) indicates the centromere position. Black crosses mark the known resistance genes at their respective physical position

*QLrYrSr.uzh-7D* covers 43.2 Mb on the long arm of chromosome 7D, residing near the pleiotropic cloned rust-resistance gene *Lr34/Yr18/Sr57* encoding an ABC transporter (Krattinger et al. 2009; Keller et al. 2018) (Fig. 4a, Supplementary Table 13). *QLrYrSr.uzh-4D* spans 108.6 Mb on the long arm of chromosome 4D, encompassing the cloned pleiotropic resistance gene *Lr67/Yr46/Sr55* encoding a hexose transporter (Moore et al. 2015) (Fig. 4b, Supplementary Table 13). The presence of the resistant haplotypes of both genes was confirmed in the population using molecular markers (Supplementary Table 14). Co-located QTLs for all three rust diseases were found in both QTL regions and leaf tip necrosis (LTN), a trait commonly linked with the presence of *Lr34/Yr18/Sr57* and *Lr67/Yr46/Sr55,* was observed in field trials.

The other two potentially novel loci confer field resistance specific to YR, and no previously described YR gene was identified within the same region. *QYr.uzh-5B* spans 29.3 Mb on the long arm of chromosome 5B and was found both in Zurich and Toluca (Fig. 4c, Supplementary Table 13). *QYr.uzh-3D.1* lies near the centromeric region of chromosome 3D, covering approximately 311.7 Mb (Fig. 4d, Supplementary Table 13). The length might be attributed to reduced recombination rates and lower marker density in the D genome. It was consistently detected in both locations, with a slightly shifted mapping position in Zurich.

### Geographic distribution of QTL effects within the NAM population

The distribution of QTL effects from combined mapping revealed that some QTLs were broadly spread (*QLrYrSr.uzh-7D* and *QYr.uzh-5B*), while others were unique in a specific family (*QLrYrSr.uzh-4D* and *QYr.uzh-3D.1*). Those patterns could also be seen when mapping each of the bi-parental families of the NAM population individually (Supplementary Figs. 13–19).

**Fig. 5:**
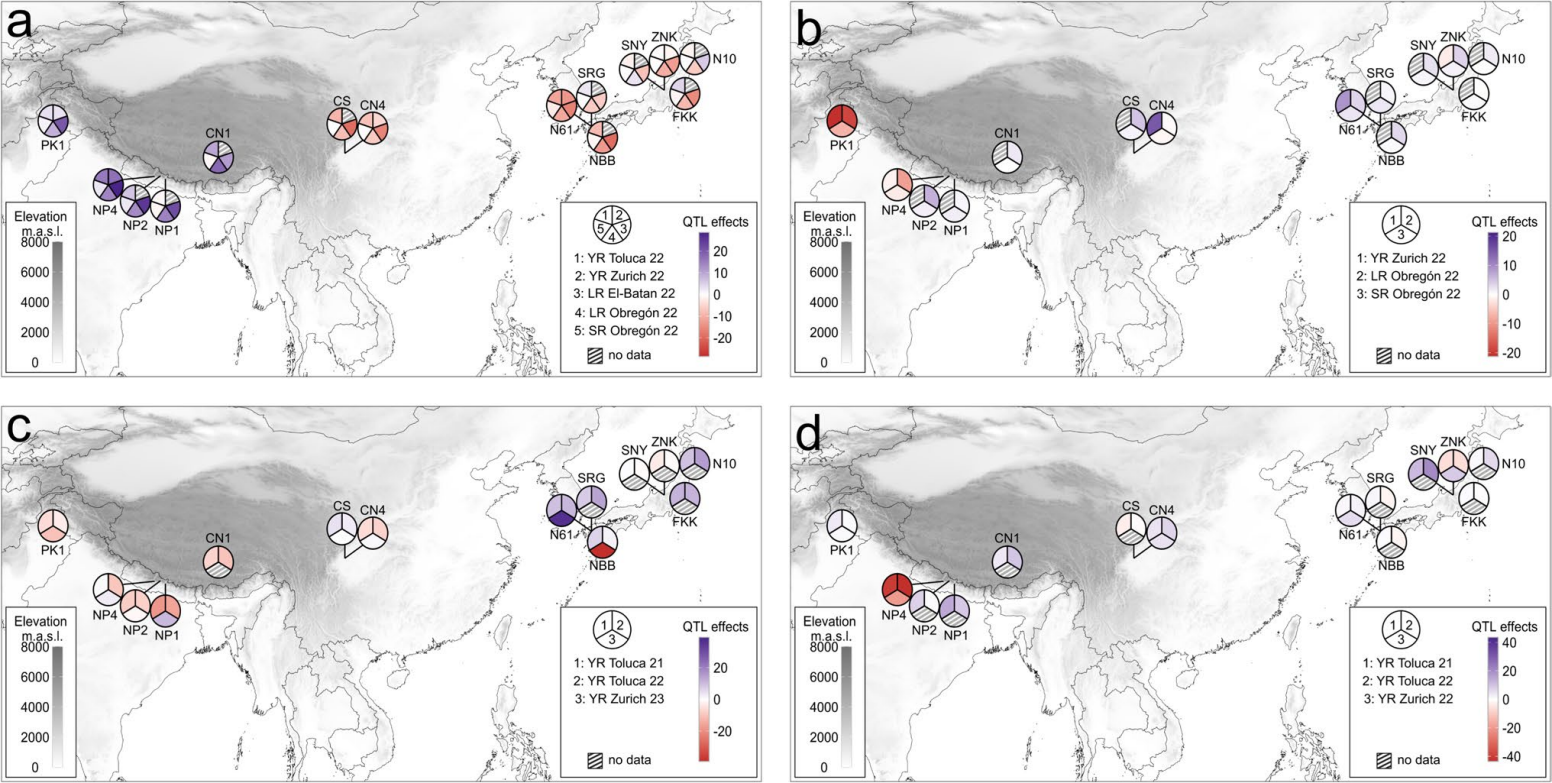
The plots represent the QTL effects of NAM families at **a** *QLrYrSr.uzh-7D*, **b** *QLrYrSr.uzh-4D*, **c** *QYr.uzh-5B*, and **d** *QYr.uzh-3D.1,* detected by combined family mapping. The color gradient of each QTL indicates the direction and strength of the effect (different color scale for each QTL), and each section of the pie chart represents one environment. Only environments with significant QTLs are represented

The effects of *QLrYrSr.uzh-7D* displayed the same two groups as the gel-based genotyping for the gene *Lr34/Yr18/Sr57* in the NAM parents (Supplementary Table 14) (Fig. 5a). On the one hand, the QTL conferred an overall resistance against mainly LR and SR to families originating from Japan, including the common parent Norin 61 and the two lines originating from the Chinese lowlands (CN4 and Chinese Spring). On the other hand, no resistant effect was found in the landrace from Tibet (CN1) and all landraces from south of the Himalayan mountains.

On *QYr.uzh-5B*, alleles with a resistance effect or an effect close to the ancestral allele were broadly distributed across regions with high disease pressure, such as south of the Himalayan Mountains and the Chinese lowlands. Additionally, a strong resistance (negative effect) on the family originating from the traditional Japanese variety NBB was found, particularly in Zurich (Fig. 5c).

The resistant effect on *QLrYrSr.uzh-4D*, which likely corresponds to *Lr67/Yr46/Sr55*, was only consistently found across all environments in the PK1 family from Pakistan (Fig. 5b). This matches the previous identification of a rust resistance in the Pakistani landrace PI 250413 (Fig. 5b, Dyck and Samborski 1979), which was later defined as *Lr67/Yr46/Sr55* (Moore et al. 2015).

Finally, we examined another potentially new locus, *QYr.uzh-3D.1*. It was present only in a traditional Nepalese variety, which exhibited strong YR resistance (Fig. 5d).

## Discussion

### Potentially new rust resistance loci

Our analysis strongly suggested that *QLrYrSr.uzh-4D* and *QLrYrSr.uzh-7D* correspond to the previously cloned pleiotropic resistance genes *Lr67/Yr46/Sr55* and *Lr34/Yr18/Sr57*, respectively. In contrast, the other two major QTLs detected in this study, *QYr.uzh-3D.1* and *QYr.uzh-5B*, possibly represent novel loci with intermediate to strong and relatively stable resistance, making them valuable resources for wheat breeding programs.

*QYr.uzh.-3D.1* spans a broad region on chromosome 3D. However, if we focus solely on the overlapping area detected in the three environments, it can be narrowed down to a range of 58 Mb on the short arm of the chromosome (Supplementary Table 15, Supplementary Fig. 20). From those QTLs we identified in our literature review, only one other locus has been previously reported to be clearly within this region: Liu et al. (2022) identified a QTL observed only in the field in Kenya, but not in Mexico. In contrast, we found the strongest evidence for *QYr.uzh-3D.1* when evaluating our NAM population at the same experimental station in Mexico (Toluca) using the same pathotype (Mex14.191). As we detected *QYr.uzh.3D.1* with a strong signal in both years in Toluca, we conclude that it represents a different locus. Three other QTLs have been reported that cover portions of our QTL region but also extend significantly beyond it. Two of them, by Basnet et al. (2014) and Lan et al. (2017), confer pleiotropic resistance, unlike *QYr.uzh-3D.1*. The third, by Cobo et al. (2018), was found in Argentinean germplasm and was only identified in one out of six environments, which does not align with the stable detection of *QYr.uzh-3D.1* across several environments. Even though *QYr.uzh-3D.1* likely represents a novel locus, further studies are needed to determine whether it differs from the previously discussed QTLs.

Several QTLs for YR resistance have been identified on chromosome 5B, forming distinct resistance clusters (Kumar et al. 2023). Most of the previously published QTLs that we found are on 5BL, of which six coincide with *QYr.uzh-5B* in our study (Supplementary Table 16, Supplementary Fig. 21). Although our current data is not sufficient to clearly determine whether they are the same or different loci, we will explain the distinct characteristics of *QYr.uzh-5B* in comparison qlr to all six below. Most NAM parents carrying *QYr.uzh-5B* were either moderately or fully susceptible at the seedling stage (Supplementary Table 17). Only PK1 exhibited high resistance; however, we believe this must be attributed to other QTLs or pleiotropic effects, as the other parents carrying *QYr.uzh-5B* did not display the same characteristic. Therefore, we assume that *QYr.uzh-5B* does not provide strong seedling resistance, contrary to the QTLs reported by Liu et al. (2020) and Feng et al. (2011), as well as the gene *Yr74* identified by Dracatos et al. (2016). Additionally, *Yr74* and *Yr73* (3DL) are complementary and confer seedling resistance only when both are present (Dracatos et al. 2016). In our data, only the NP4 family carries both a QTL on chromosome 5B (*QYr.uzh-5B*) and another on chromosome 3D (*QYr.uzh-3D.1*). While *QYr.uzh-5B* overlaps with *Yr74*, *QYr.uzh-3D.1* is ~ 100 Mb from *Yr73*. *QYr.uzh-3D.2* is closer to *Yr73*, but none of our genotypes harbor both *QYr.uzh-5B* and *QYr.uzh-3D.2* (Supplementary Table 17). Two QTLs by Feng et al. (2011), mentioned above, and Lu et al. (2009) cover an extensive physical range greater than 200 Mb. As they are located in a region that encompasses several resistance gene clusters (Kumar et al. 2023), it is difficult to confirm if they encompass the same loci as *QYr.uzh-5B*. The QTL by Lu et al. (2009) was described as a minor QTL derived from the Italian wheat cultivar Libellula, which was observed in only one environment with a very low LOD score. A QTL for yellow rust resistance was found in the UK winter wheat line NSA-98–0995 (Vazquez et al. 2015), which was also linked to Cephalosporium stripe resistance. In our study, we did not observe any pleiotropic resistance to leaf or stem rust. However, this does not rule out the possibility that *QYr.uzh-5B* could have a pleiotropic effect on other diseases. Finally, a 3.7 Mb QTL region on chromosome 5B from Chinese landraces, identified in two studies (Long et al. 2021; Deng et al. 2022), overlaps with *QYr.uzh-5B*, with TraesCS5B02G355100 suggested as a candidate gene (Long et al. 2024). The diagnostic marker for this QTL did not match resistance patterns in our NAM population (Supplementary Table 14). However, it has been reported that this marker is not reliably diagnostic, and our results should be interpreted with caution. Further testing is necessary to determine whether the identified locus is distinct or not. If the reported QTL corresponds with *QYr.uzh-5B*, it would further support the notion that this locus is widely distributed in high-disease pressure environments and could serve as a highly durable, promising resource for resistance breeding. In summary, while we consider *QYr.uzh-5B* as a potentially novel locus here, follow-up studies are essential to definitively confirm whether it is distinctly different from the previously mentioned overlapping loci.

Three out of the four main yellow rust resistance loci detected in this study were located on the D subgenome: *QYr.uzh.3D.1* as well as two previously known loci (*Lr67/Yr46/Sr55* on chromosome 4D and *Lr34/Yr18/Sr5* on 7D). The D subgenome of bread wheat was derived from the wild wheat *Aegilops tauschii*, and it is thought to have contributed to the range expansion of hexaploid bread wheat (AABBDD genome) compared with tetraploid wheat (AABB genome) through tolerance to broad abiotic environments (Dubcovsky and Dvorak 2007; Shimizu 2022; Okada and Shimizu 2024). The D subgenome was also suggested to confer blast disease resistance as an adaptive advantage (Asuke et al. 2021). Consistent with this idea, a new wheat blast resistance gene was found in the D subgenome ancestor of *Ae. tauschii* (Yoshioka et al. 2024). This corroborates the importance of the D subgenome in resistance to biotic stresses of bread wheat. The study of yellow rust resistance of synthetic bread wheat derived from natural accessions of *Ae. tauschii* (the DD genome) revealed many resistance accessions, suggesting further opportunities to enhance the resistance in the D subgenome (Kishii et al. 2019).

The NAM lines containing the two potentially novel loci could be directly selected and incorporated into breeding programs. Since these lines were crossed with the modern cultivar Norin 61, they are less likely to cause substantial linkage drag, as commonly seen when working with traditional varieties.

### Distribution of resistance loci within the NAM population

Analyses of the family-wise effects revealed two types of QTLs in this study: those containing QTLs exclusively in a single NAM family (such as *QYr.uzh-3D.1* and *QLrYrSr.uzh-4D*) and those with QTLs broadly distributed over several NAM families (such as *QLrYrSr.uzh-7D* and *QYr.uzh-5B*). First, we discuss the two main QTLs that were broadly distributed within the NAM population.

The effects of *QLrYrSr.uzh-7D*, now referred to as *Lr34/Yr18/Sr57*, reveal a clear separation between varieties from the east of the Himalayan mountains and those from the south. This observation matches previous reports about the presence of the resistant allele of this gene: Kolmer et al. (2008) suggested that *Lr34/Yr18/Sr57* originated from a traditional Chinese variety, which was later supported by Dakouri et al. (2014), and previous work indicates that wheat might have first been introduced to Japan from China (Nakamura 2002; Tanaka et al. 2005; Okada and Shimizu 2024). The clear separation could potentially be explained by the high Himalayan mountain ranges acting as a physical barrier for gene flow between its two sides, as also described in other studies focusing on naturally occurring plant and mushroom species in this region (Feng et al. 2017; Qiong et al. 2017). The broad distribution of the resistance-conferring haplotype of this gene in our population suggests that it confers highly durable resistance, consistent with previous reports (Singh 1992).

Another example of a resistance locus present in several NAM families is *QYr.uzh-5B*, identified in varieties from both sides of the Himalayan mountains and all high-disease pressure areas. Despite facing strong and constantly changing disease pressure, the resistant haplotype of *QYr.uzh-5B* is distributed widely, suggesting it could be a highly durable source of YR resistance. Additionally, only the NBB family contributes to a strong location-specific resistance on *QYr.uzh-5B*, displaying unique characteristics that might result from a different underlying gene or haplotype. This is not unlikely, given several resistance gene clusters on chromosome 5BL (Kumar et al. 2023). However, this observation is based on data from just one year. Follow-up trials are necessary to determine whether this resistance remains stable over time.

The broad distribution of *QLrYrSr.uzh-7D* and *QYr.uzh-5B* could imply that traditional varieties from these critical regions are essential in conserving highly durable alleles of YR resistance. Narrowing down the genomic region and cloning *QYr.uzh-5B* could help to better understand the molecular basis of durable resistances. It was shown recently that *Lr34/Yr18/Sr57* increases the thickness of the cell wall and protects the plants from the pathogen by transporting sinapyl alcohol through the cell wall (Zhang et al. 2024). Following up on the loci discovered in our study could help to find out whether resistance genes that are broadly distributed and seemingly adapted to high disease pressure share similar molecular mechanisms.

We also observed two loci (*QLrYrSr.uzh-4D* and *QYr.uzh-3D.1*) that were unique in a single variety within the NAM population. *QLrYrSr.uzh-4D*, covering the gene *Lr67/Yr46/Sr55*, was found exclusively in the Pakistani PK1 family, aligning with the gene’s discovery region (Moore et al. 2015). Meanwhile, strong resistance on *QYr.uzh-3D.1* was contributed solely by the Nepalese NP4 family. Further testing in a more significant number of traditional varieties would be needed to confirm if the frequency of these resistance alleles are low, or they were by chance not covered by our population in traditional varieties from further geographic areas. Their potential uniqueness might be explained by an adaptation of the host plant to the different YR populations in the near-Himalayan region that exhibit limited gene flow between each other (Hu et al. 2017; Khan et al. 2019). Some barriers in this geographic region seem to separate the YR populations, possibly due to geography, ecological constraints, and cultural practices. The barriers that hinder the gene flow between YR populations might also limit the distribution of these resistance loci.

Both QTLs discussed here (*QLrYrSr.uzh-4D* and *QYr.uzh-3D.1*) conferred resistance in the two tested locations, Mexico and Switzerland, suggesting their potential to provide valuable resistance beyond their adapted environments. Notably, the gene *Lr67/Yr46/Sr55* has already been reported to confer resistance across multiple regions, for example, North-West Europe (Zelba et al. 2024). This could make these loci valuable potential resources for breeding programs in diverse areas.

### Strong expression of resistances due to the combination of multiple resistance genes

The traditional wheat lines from the near-Himalayan region and the Chinese lowland tested in this study show stable resistances, close to immunity, likely due to the combination of several resistance genes. Similar observations were reported previously, for example, in the cultivars Francolin #1 (Lan et al. 2014) or Kundan (Ren et al. 2017). The observed level of resistance loci can be influenced by genotype-by-environment interactions or specificity to different rust races, which can lead to varying levels of resistance as reported in the variety Borlaug 100 (Ye et al. 2022). Stacking several resistance genes has been suggested as a promising strategy for achieving more durable long-term disease resistance, compared to focusing solely on race-specific R genes that are easier to overcome by the pathogen (Bouvet et al. 2022; Wang et al. 2023). This approach might be particularly advantageous for host plants adapted to environments characterized by high disease pressure and the ongoing appearance of new pathotypes, which might favor the accumulation of different resistance genes in traditional varieties. Although only the main effect QTLs of our study were highlighted, several small-effect APR genes are probably present in the population that explain more of the missing phenotypic variation and can be observed as more minor, non-significant peaks in our data. Follow-up studies with larger population sizes within promising families and less conservative significant thresholds could help to understand the combination of several resistance genes better, including those with minor effects. This study’s QTLs associated with yellow rust resistance represent valuable resources for breeding varieties with potentially long-term rust resistance.

### Unique genetic resources from areas with high yellow rust disease pressure

We identified different QTLs conferring YR resistance in all our tested NAM families from south of the Himalayan Mountains and the Chinese lowlands, areas characterized by high disease pressure. Even though these results are based on a few cultivars that could not capture the whole diversity present in this region, they might hint at a prevalence of YR resistance in the unique germplasm from this geographic region. Follow-up studies screening a broader panel of diverse traditional wheat lines would be needed to confirm these observations. Nevertheless, this idea is supported by cumulative evidence from earlier studies: a high density of YR-resistant wheat genotypes was observed by Maccaferri et al. (2015), and Kumar et al. (2016), and several resistance loci have been detected in traditional varieties from China (Lan et al. 2010; Ma et al. 2015; Wu et al. 2016; Yuan et al. 2018; Gessese et al. 2019; Wang et al. 2019; Long et al. 2019; Pang et al. 2022), Pakistan (Bux et al. 2012; Khalid et al. 2020; Tariq et al. 2021), and India (Chhetri et al. 2016; Raghu et al. 2018). Even though large global collections of traditional wheat varieties, such as the A. E. Watkins landrace collection or the Vavilov wheat diversity panel, have been screened for new resistance loci, they did not capture many traditional varieties from south of the Himalayan mountains (Jambuthenne et al. 2022; Cheng et al. 2024). Therefore, this NAM population can be seen as a complementary resource, focusing on a critical geographic yellow rust hot spot.

Our findings suggest that the near-Himalayan region is a critical reservoir of genetic diversity and rust resistance, and two complementary factors could explain the phenomenon. First, the high biodiversity that is driven by various climates, elevations, and heterogeneous environments in the Himalayan mountains (Wambulwa et al. 2021). This may have contributed to the rust resistance patterns observed in our study and the unique variation in traditional wheat varieties (Balfourier et al. 2019; Lehnert et al. 2022). A second factor is the historical co-existence with yellow rust. The Himalayan region has been known for wheat cultivation for over 3500 years (Stevens et al. 2016), is recognized as the geographic origin of the YR pathogen, and has, therefore, experienced significant disease pressure (Ali et al. 2014, 2017; Thach et al. 2016; Hu et al. 2017; Khan et al. 2019; Li et al. 2021a). This co-existence, combined with selective practices by farmers, likely led to the high diversity of traditional wheat varieties adapted to such conditions. This scenario implies that preserving traditional wheat varieties in genebanks and through on-field conservation practices in regions with high disease pressure would be crucial to counteract genetic erosion, leverage the genetic potential for breeding YR-resistant cultivars, and enable benefit-sharing with local communities (Bellon et al. 2017; Khoury et al. 2022).

## Conclusion

Our study highlights the importance of traditional wheat varieties in providing YR resistance in wheat, with a focus on resistance in the near Himalayan region. This emphasizes the pivotal role of local farmers in conserving and enhancing genetic diversity. We identified two potentially new resistance loci on chromosome 3D (*QYr.uzh-3D.1*) and 5B (*QYr.uzh-5B*), which could serve as a valuable resource for modern breeding efforts. The broad distribution of *QYr.uzh-5B* within our NAM population suggests it to be a promising candidate for further research to understand the long-term durability of resistance under strong disease pressure. By utilizing a NAM population coupled with advanced data analysis and leveraging recent advancements in reference genome quality, our study provides valuable insights into rust resistance in wheat in a critical geographic area.

## Supplementary Information

Below is the link to the electronic supplementary material.

Supplementary file1 (PDF 3298 KB)

Supplementary file2 (XLSX 214 KB)

Supplementary file3 (XLSX 22198 KB)

Supplementary file4 (R 6 KB)

## Data Availability

All input files used for QTL mapping can be found in the Supplementary file 1, including phenotypes and genotypes of all families and the consensus map. The R code used for QTL mapping can be found in Supplementary file 2. Seeds of the 25 parental lines of the NAM population, as well as the NAM population itself, can be retrieved from the National BioResource Project under the following link: http://www.shigen.nig.ac.jp/wheat/komugi/.
