# Supplementary material for: Unveiling yellow rust resistance in the near-Himalayan region: insights from a nested association mapping study
Source: Theor Appl Genet. 2025 Jun 5;138(7):135. doi: 10.1007/s00122-025-04886-z (PMC12141412; doi:10.1007/s00122-025-04886-z)
Supplement: Supplementary file 1 — Supplementary file1 (PDF 3298 KB) [file 122_2025_4886_MOESM1_ESM.pdf]

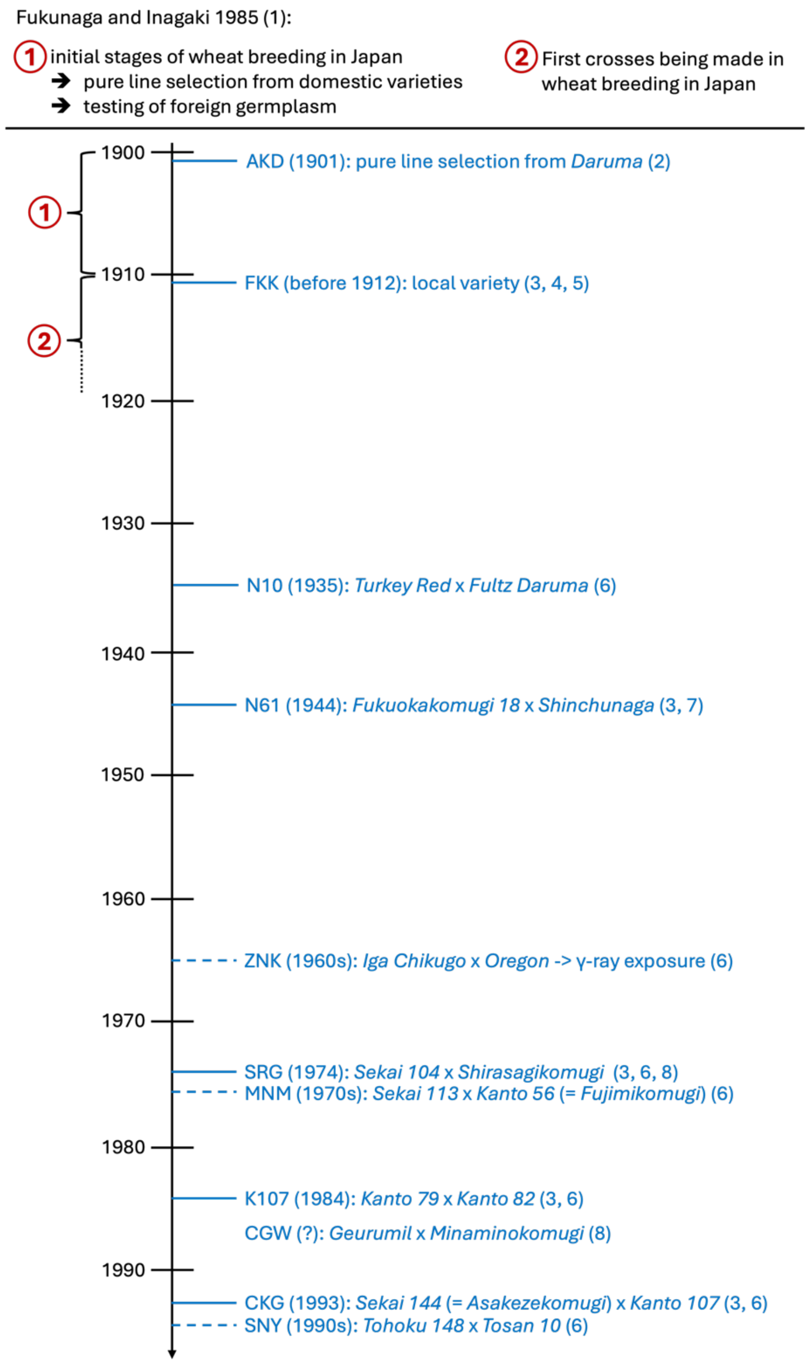

(1) Fukunaga K, Inagaki M (1985) Genealogical pedigree of Japanese wheat cultivars. Japanese J Breed 35:89–92

(2) Noda K (1986) Daruma and Akakomugi. Seiken Ziho, Rep Kihara Inst Biol Res 34:1–14

(3) Kojima H, Fujita M, Matsunaka H, Seki M, Chono M, Kiribuchi-Otobe C, Takayama T, Oda S (2017) Development and Evaluation of the Core Collection of Japanese Wheat Varieties. Bull NARO Crop Sci 1:1–13

(4) NARO (2024) Details of plant genetic resources. https://www.gene.affrc.go.jp/databases-plant_search_detail_en.php?jp=20691. Accessed 22 Jan 2024

(5) Zeven AC, Zeven-Hussink NC (1976) Genealogies of 14.000 wheat varieties. Wageningen

(6) Hisashi Yoshida (2021) Nihon no komugi touroku hinshu no keifu - hinshu kaihatsu no 100 nen -. (in Japanese) Agriculture and horticulture, 96:7, 579-586.

(7) NARO (2024) Japanese Wheat Core Collection. https://www.gene.affrc.go.jp/databases-core_collections_jw.php. (KOMUGI NOURIN 61) Accessed 22 Jan 2024

(8) Naoyuki Ishikawa (2003) 2-2 Ondanchi ni okeru komugi no wasekaikushu no genjo to tenbo. (in Japanese) Fuyusakumotsukenkyu 3: 34-38.

Supplementary Figure 1: Timeline showing the year of development and pedigree of Japanese NAM parents.

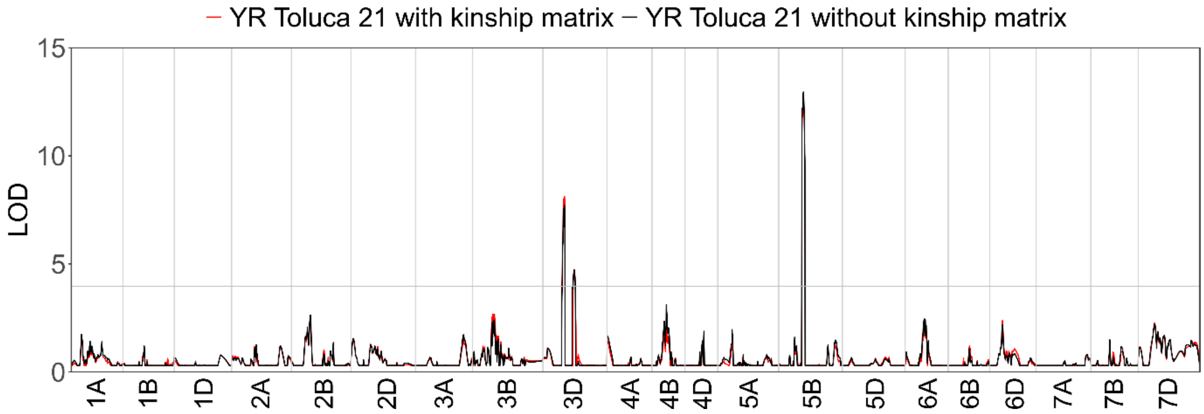

Supplementary Figure 2: Comparison of single QTL mapping for yellow rust in Toluca 2021 with and without the use of a kinship matrix

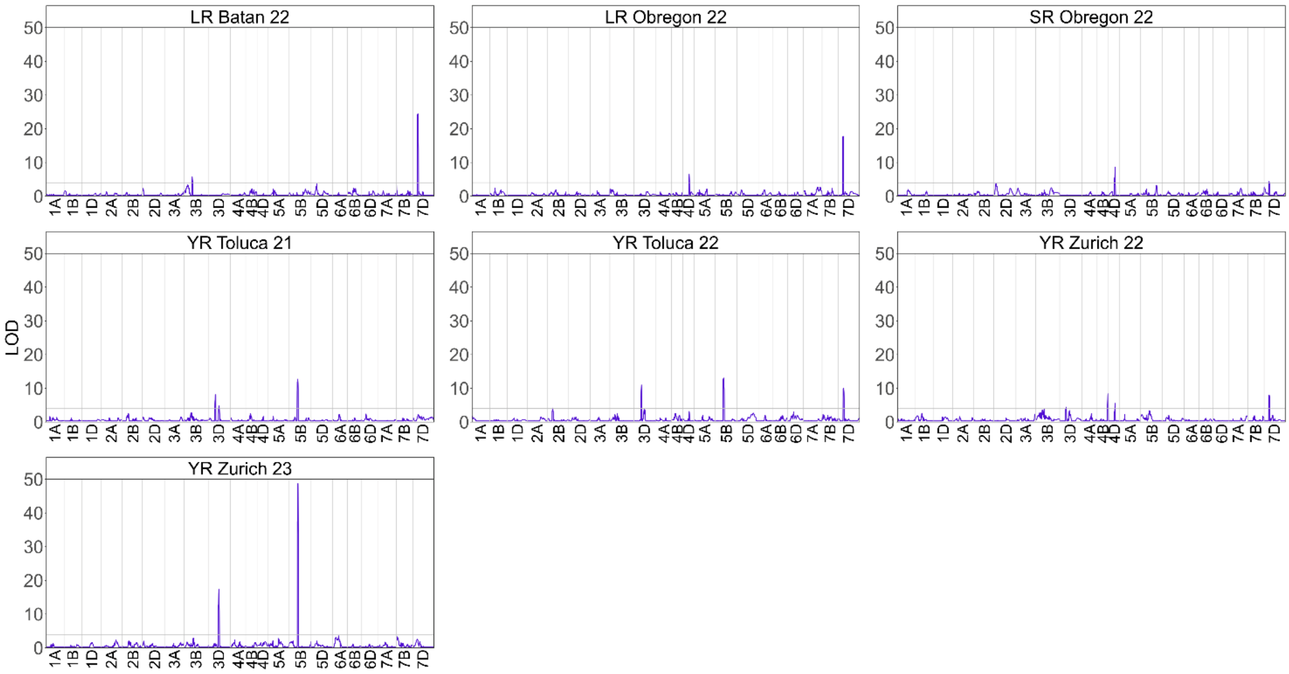

Supplementary Figure 3: Combined QTL mapping for all families in seven different environments.

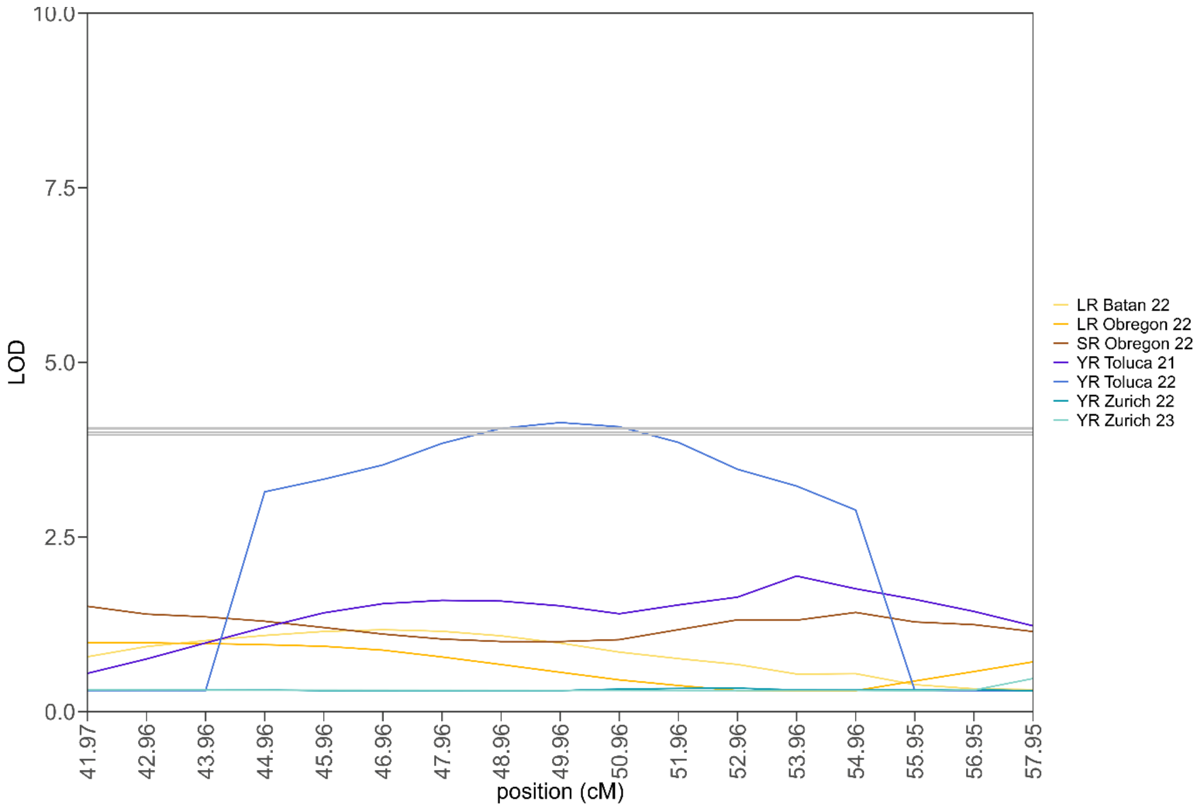

Supplementary Figure 4: Detailed representation of *QYr.uzh-2B*

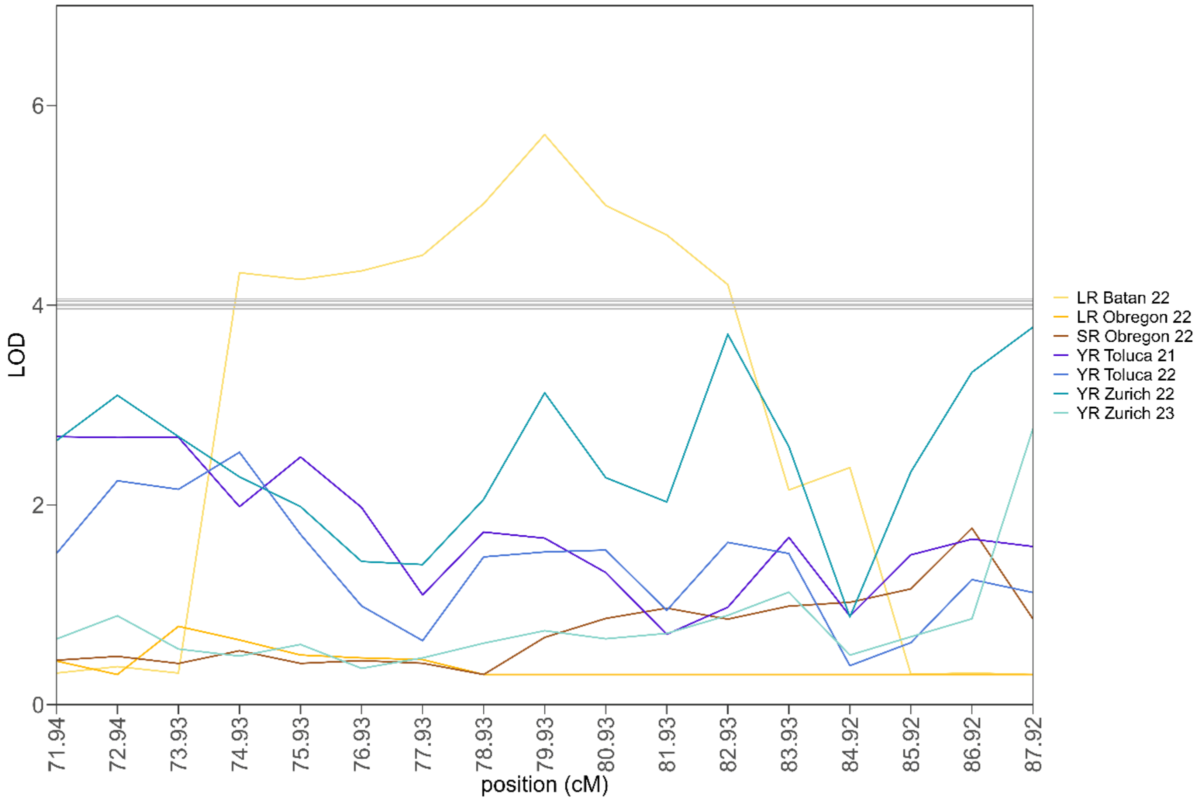

Supplementary Figure 5: Detailed representation of *QLr.uzh-3B*

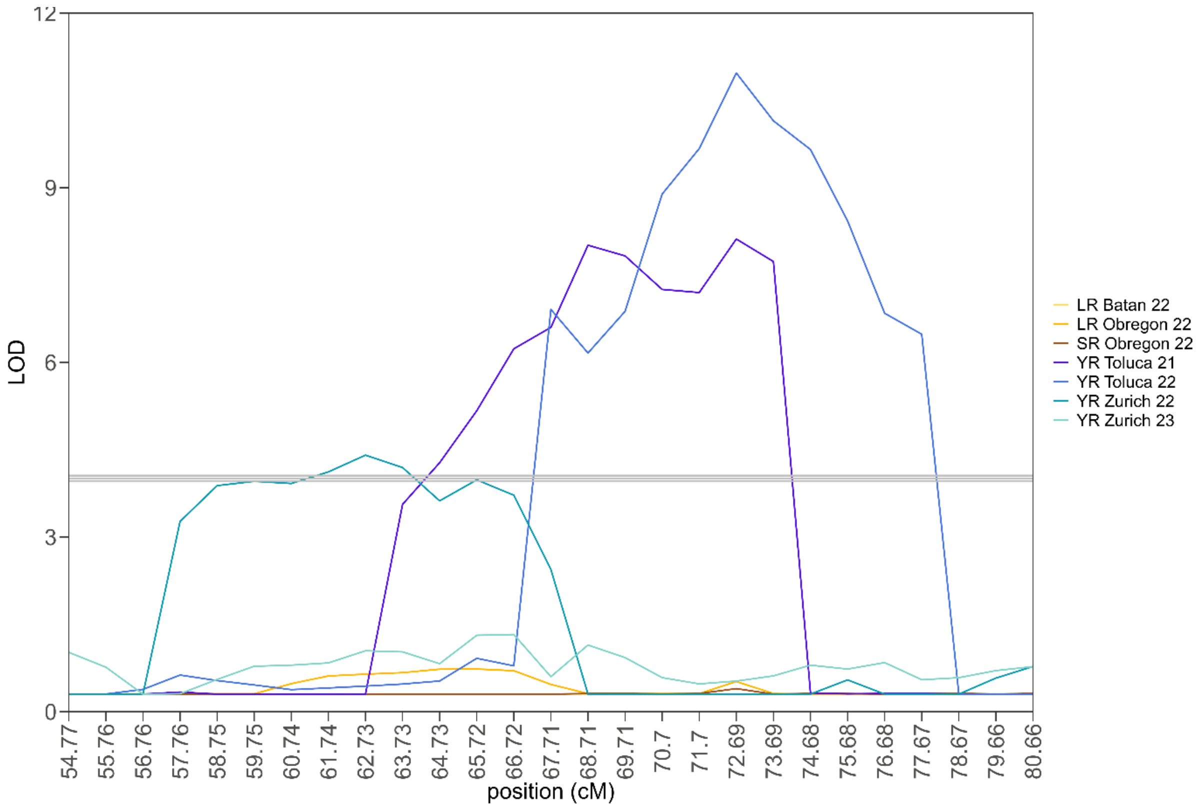

Supplementary Figure 6: Detailed representation of *QYr.uzh-3D.1*

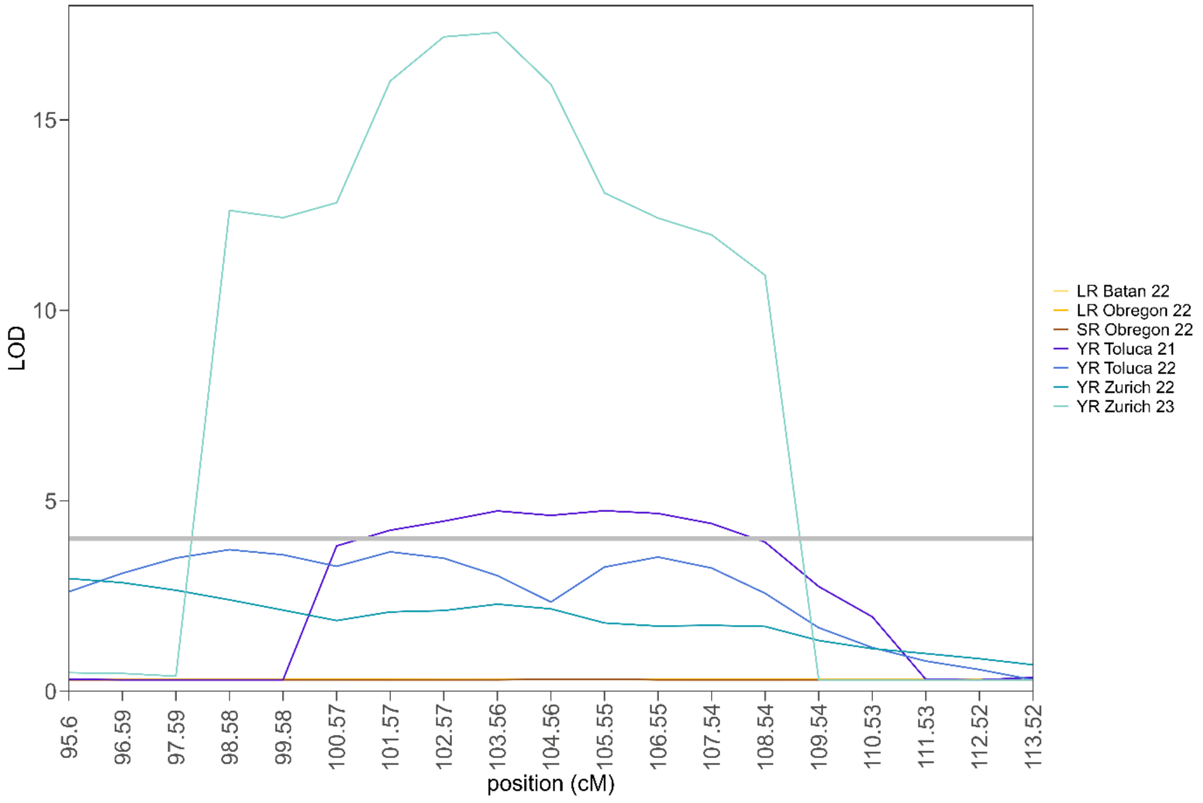

Supplementary Figure 7: Detailed representation of *QYr.uzh-3D.2*

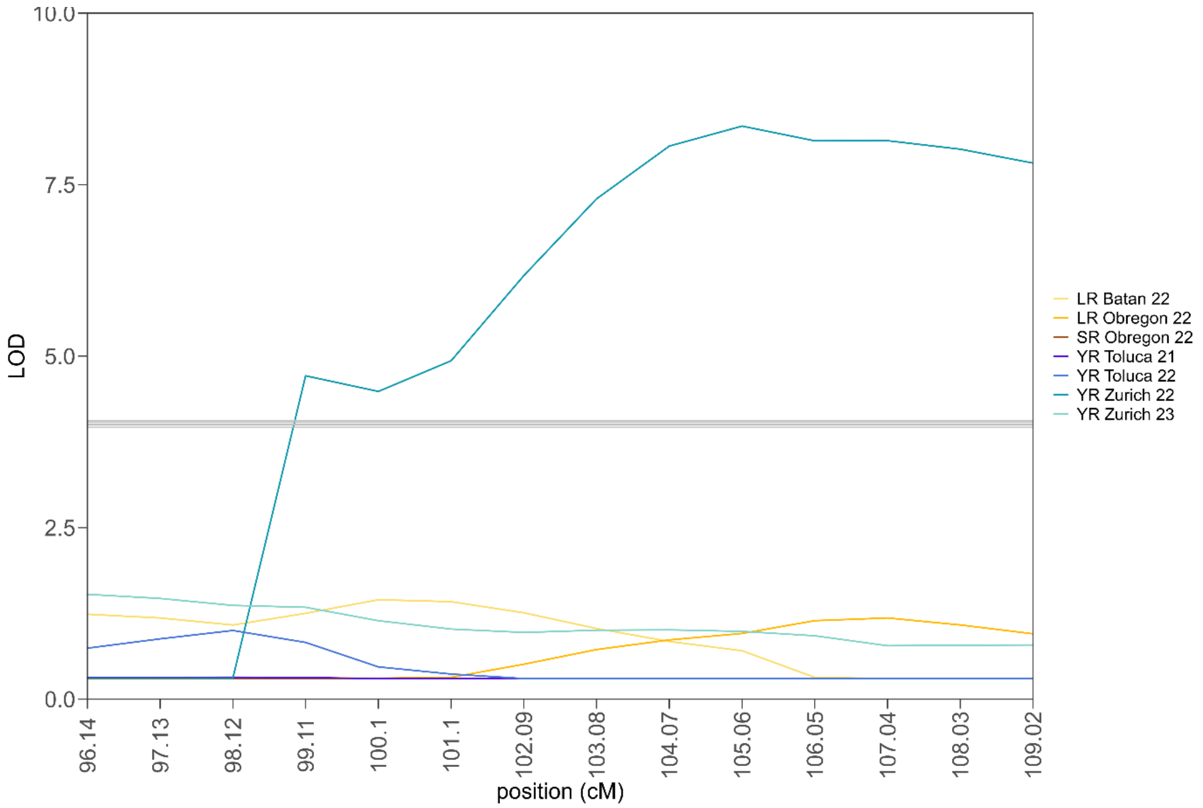

Supplementary Figure 8: Detailed representation of *QYr.uzh-4B*

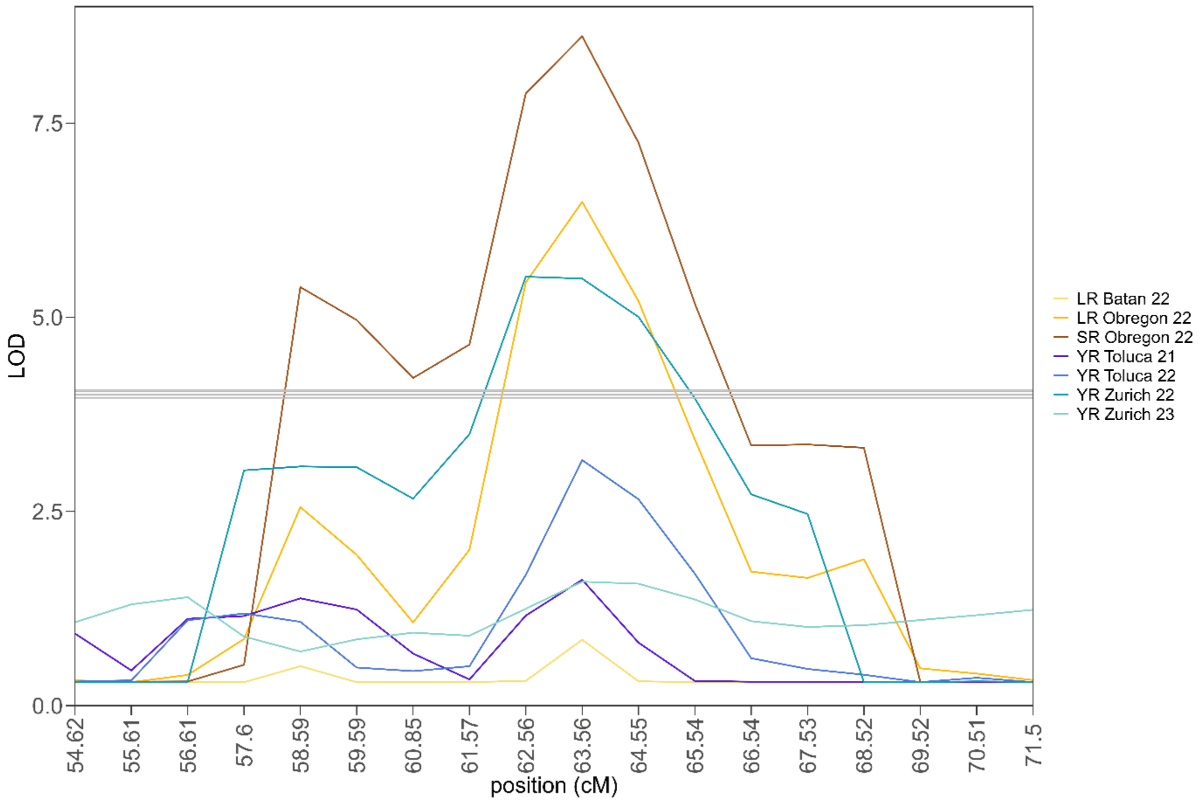

Supplementary Figure 9: Detailed representation of *QLrYrSr.uzh-4D*

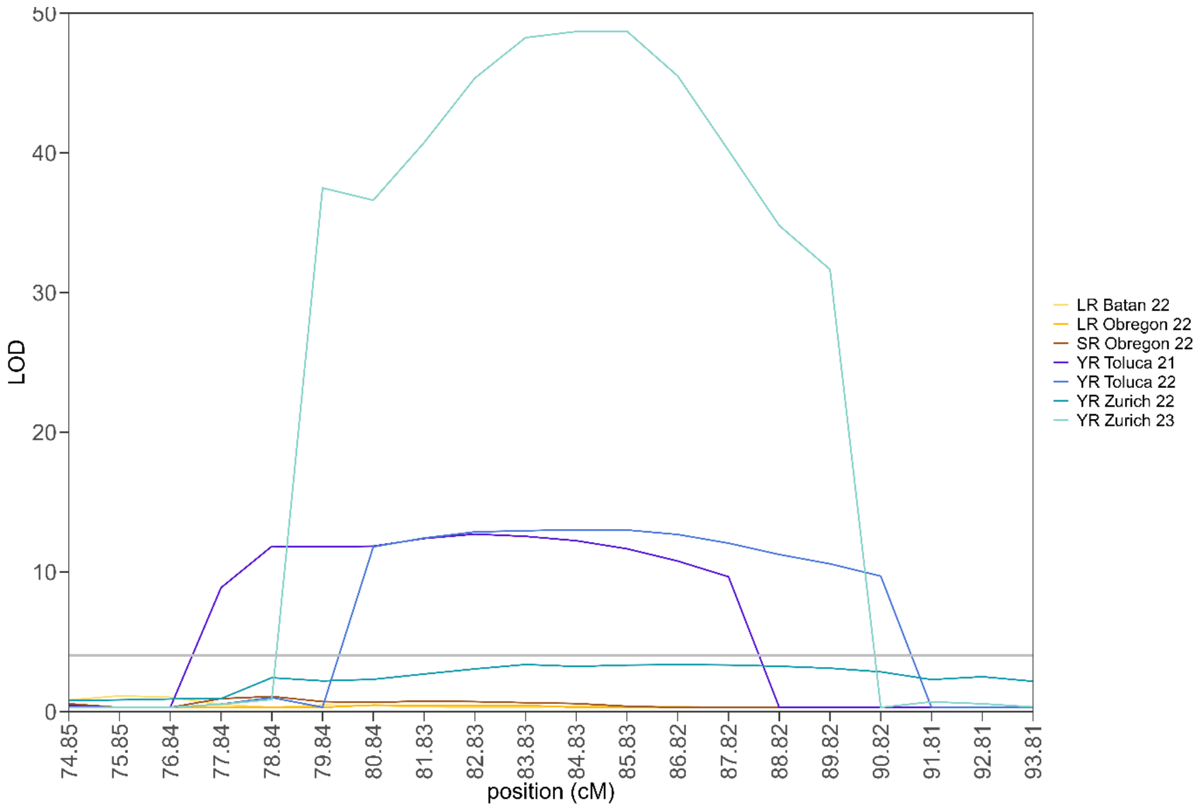

Supplementary Figure 10: Detailed representation of *QYr.uzh-5B*

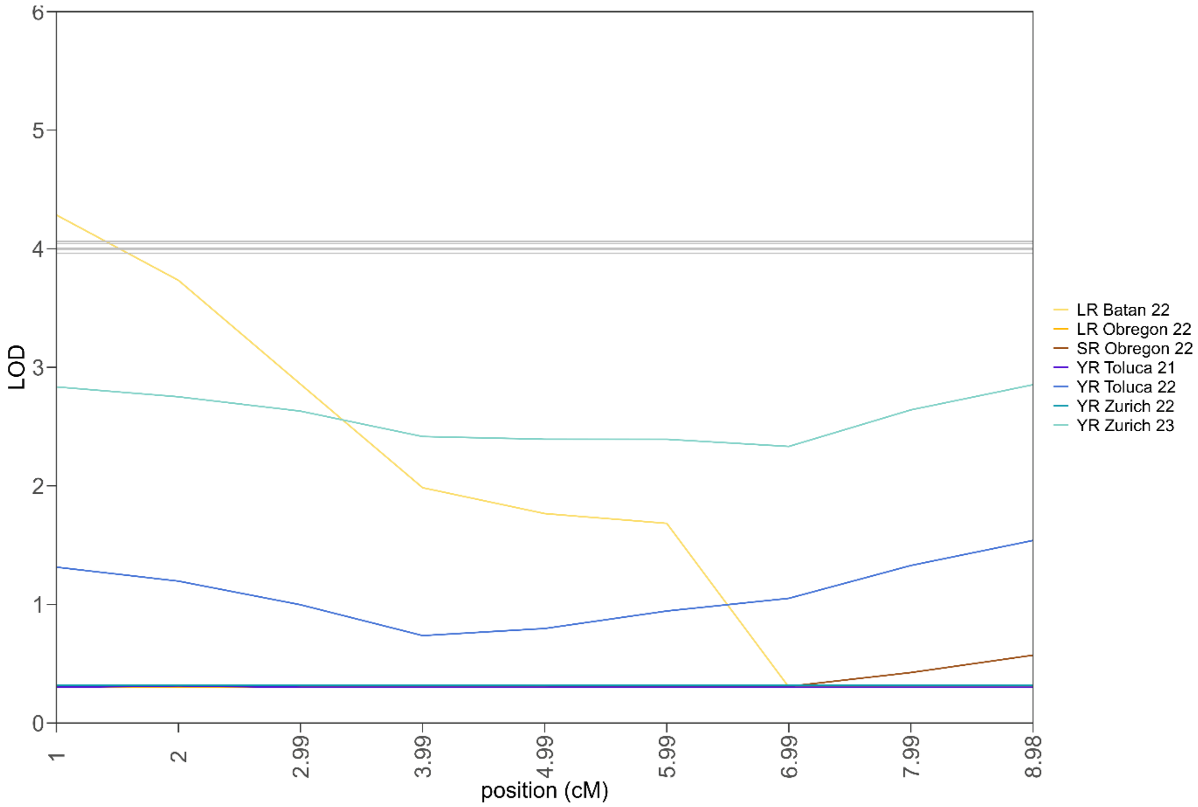

Supplementary Figure 11: Detailed representation of *QLr.uzh-7B*

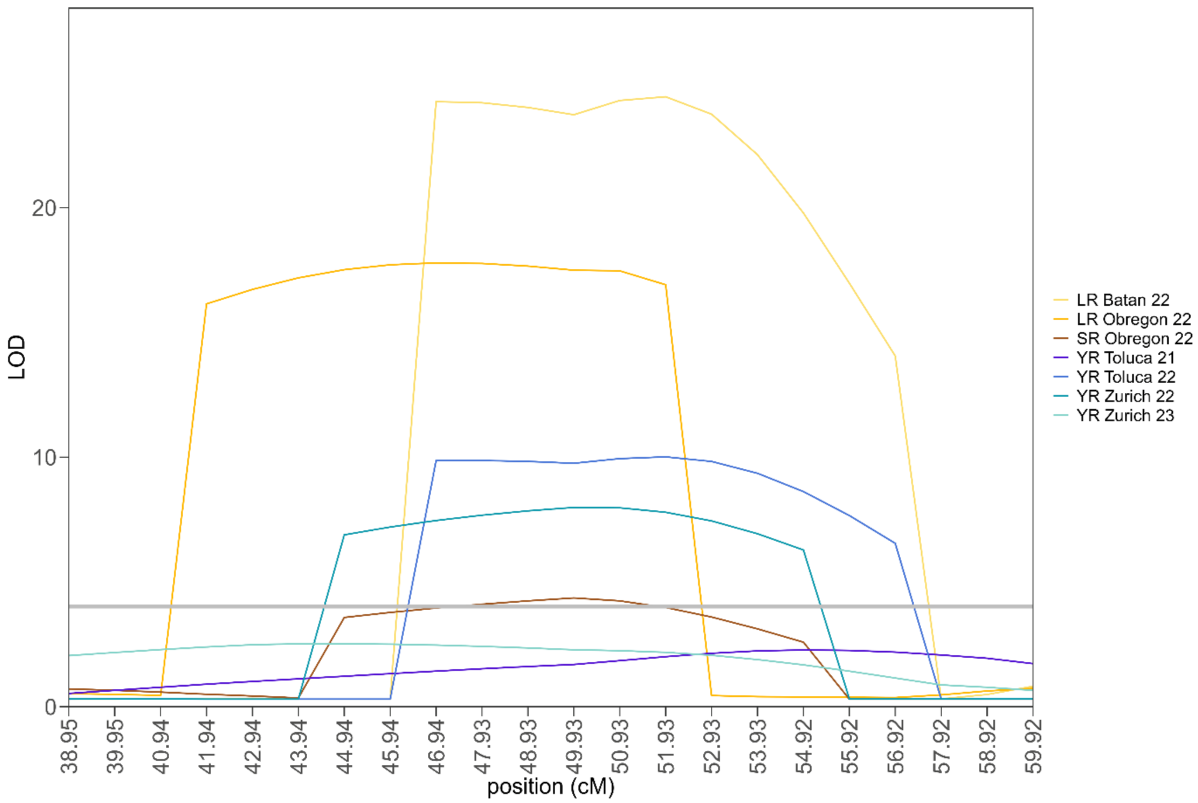

Supplementary Figure 12: Detailed representation of *QLrYrSr.uzh-7D*

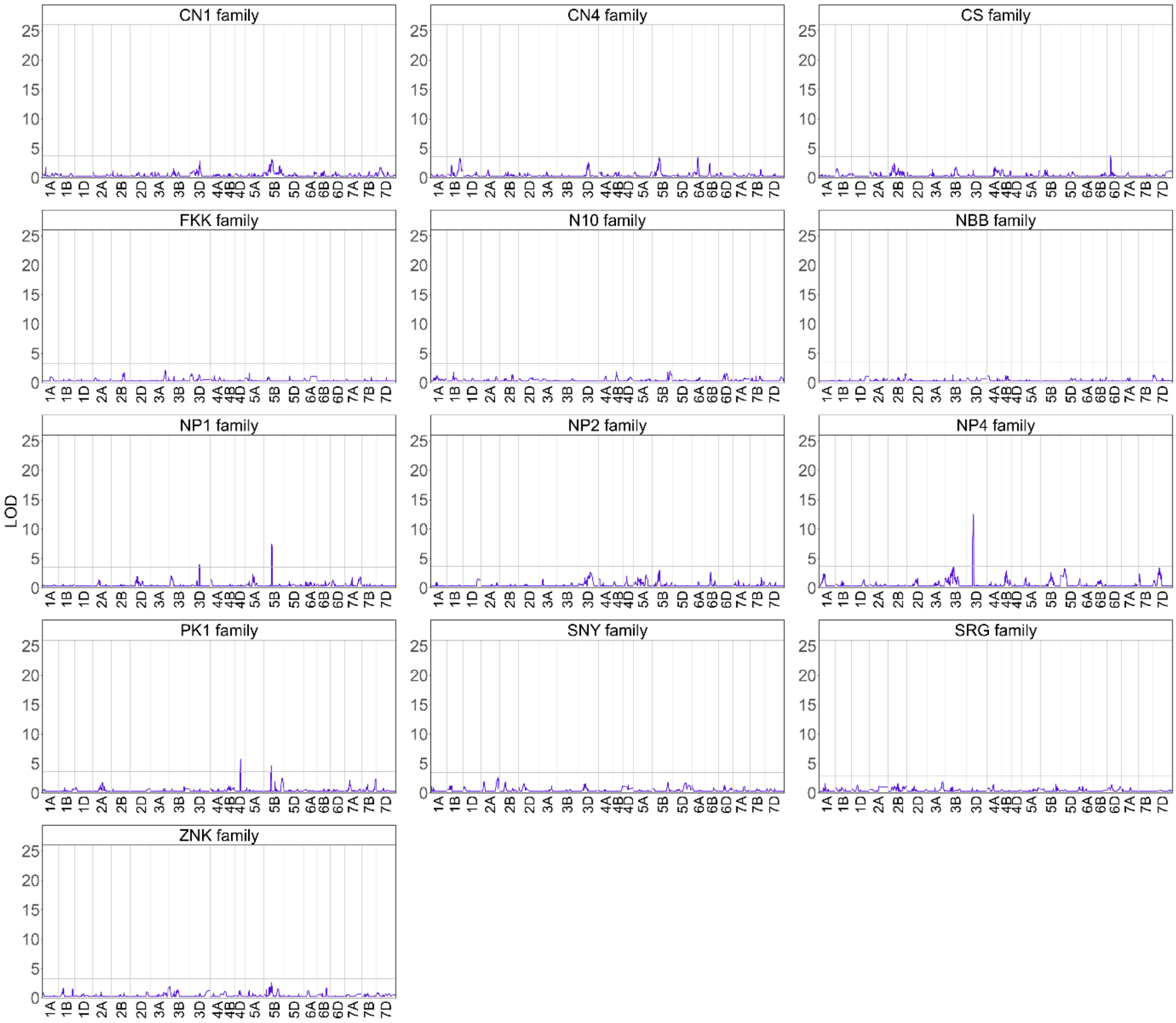

Supplementary Figure 13: Single-family QTL mapping for yellow rust data collected in Toluca 21.

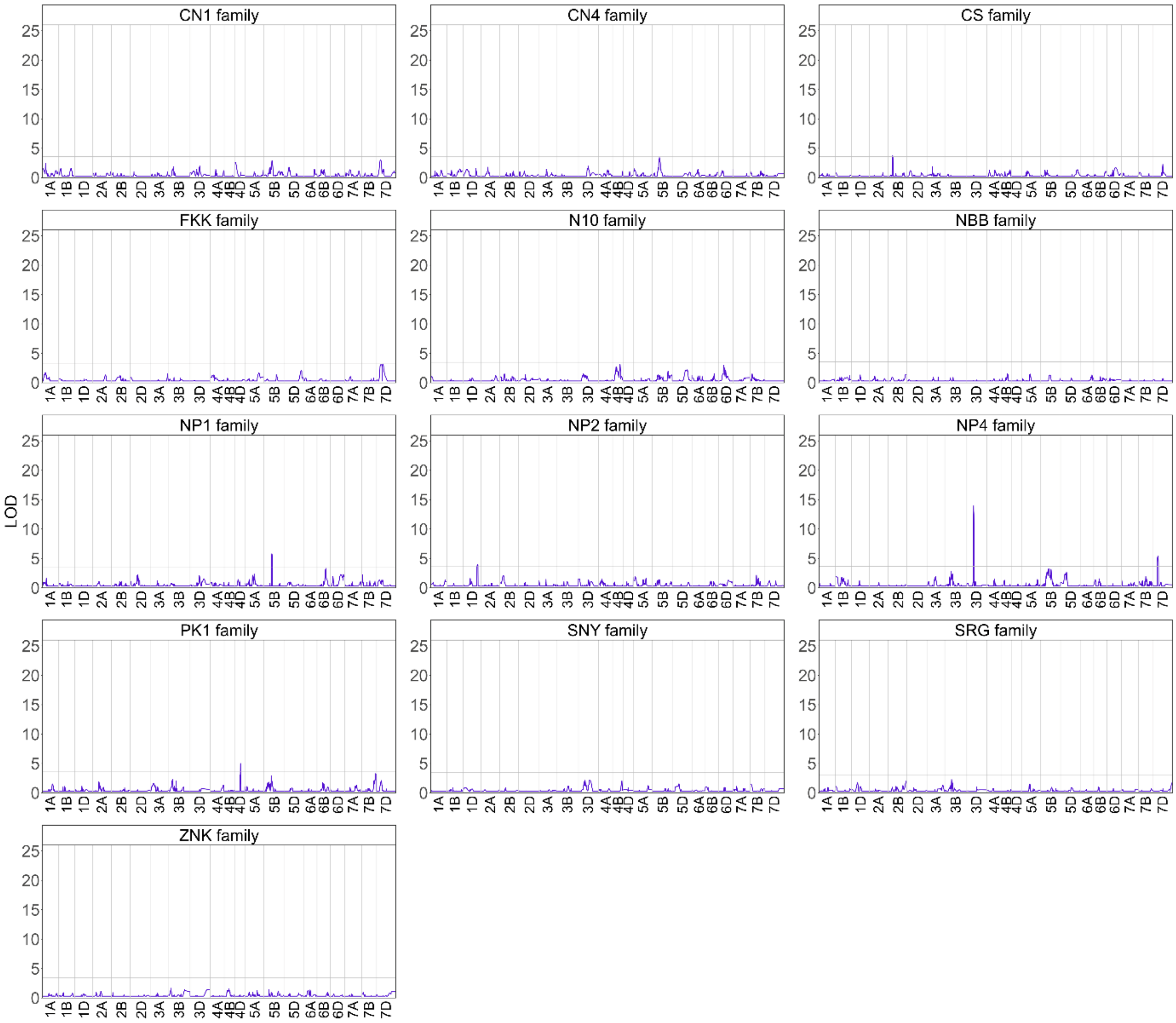

Supplementary Figure 14: Single-family QTL mapping for yellow rust data collected in Toluca 22.

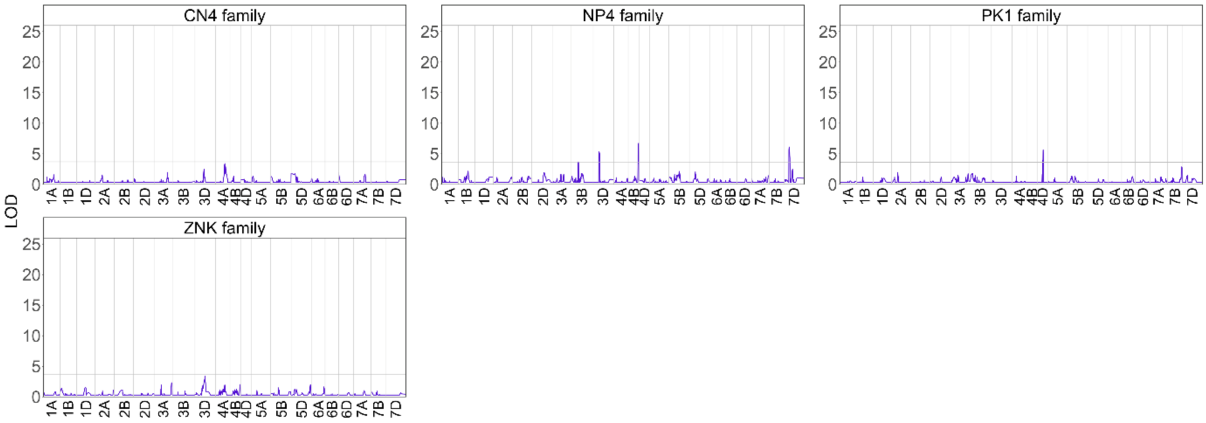

Supplementary Figure 15: Single-family QTL mapping for yellow rust data collected in Zurich 22.

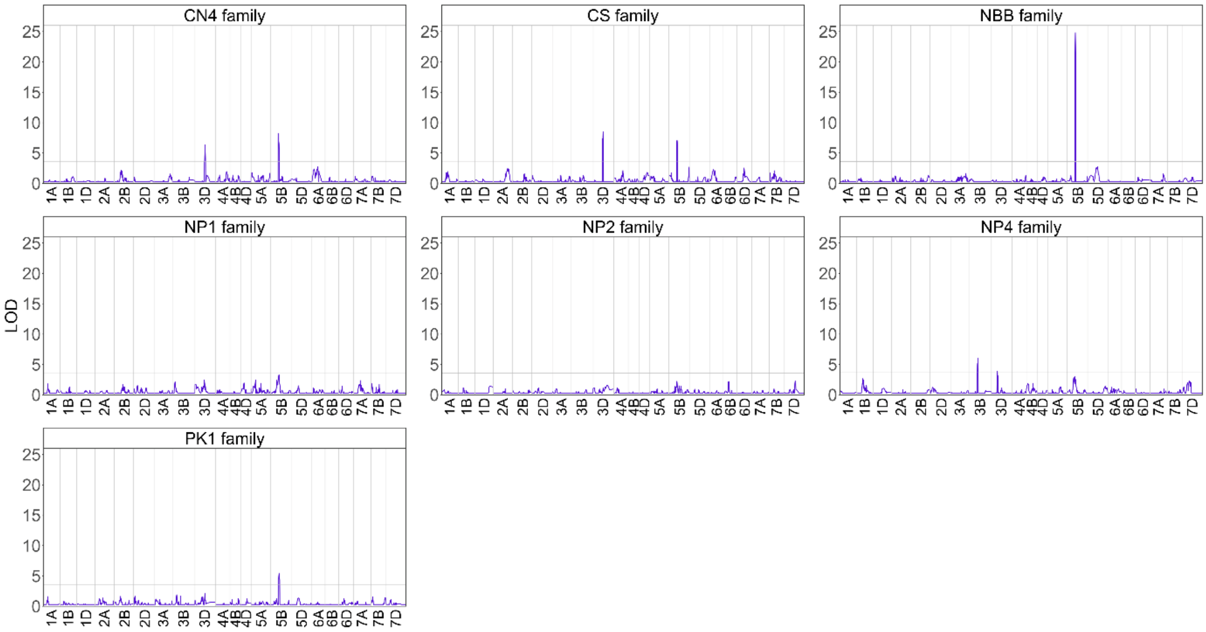

Supplementary Figure 16: Single-family QTL mapping for yellow rust data collected in Zurich 23.

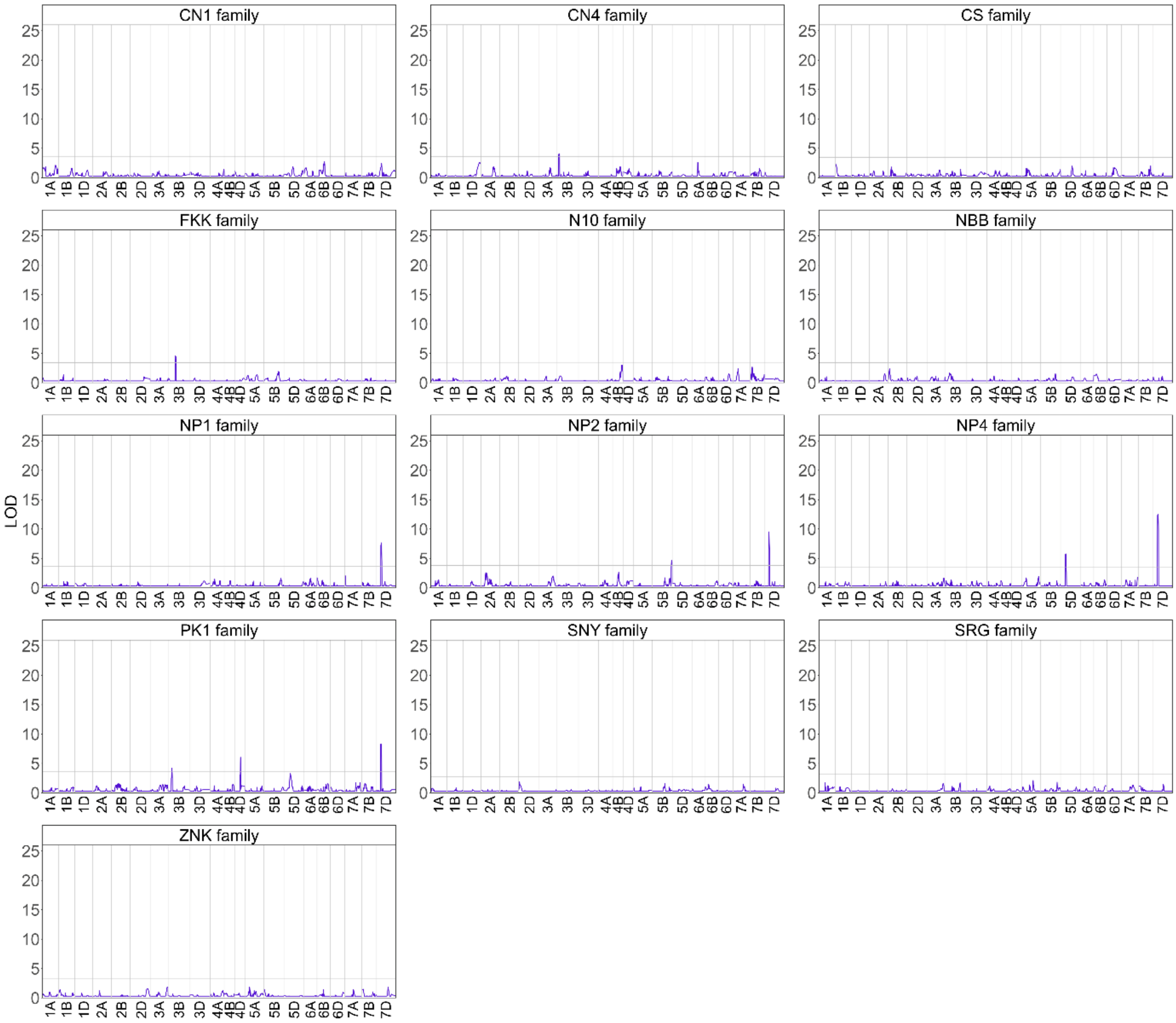

Supplementary Figure 17: Single-family QTL mapping for leaf rust data collected in El-Batan 22.

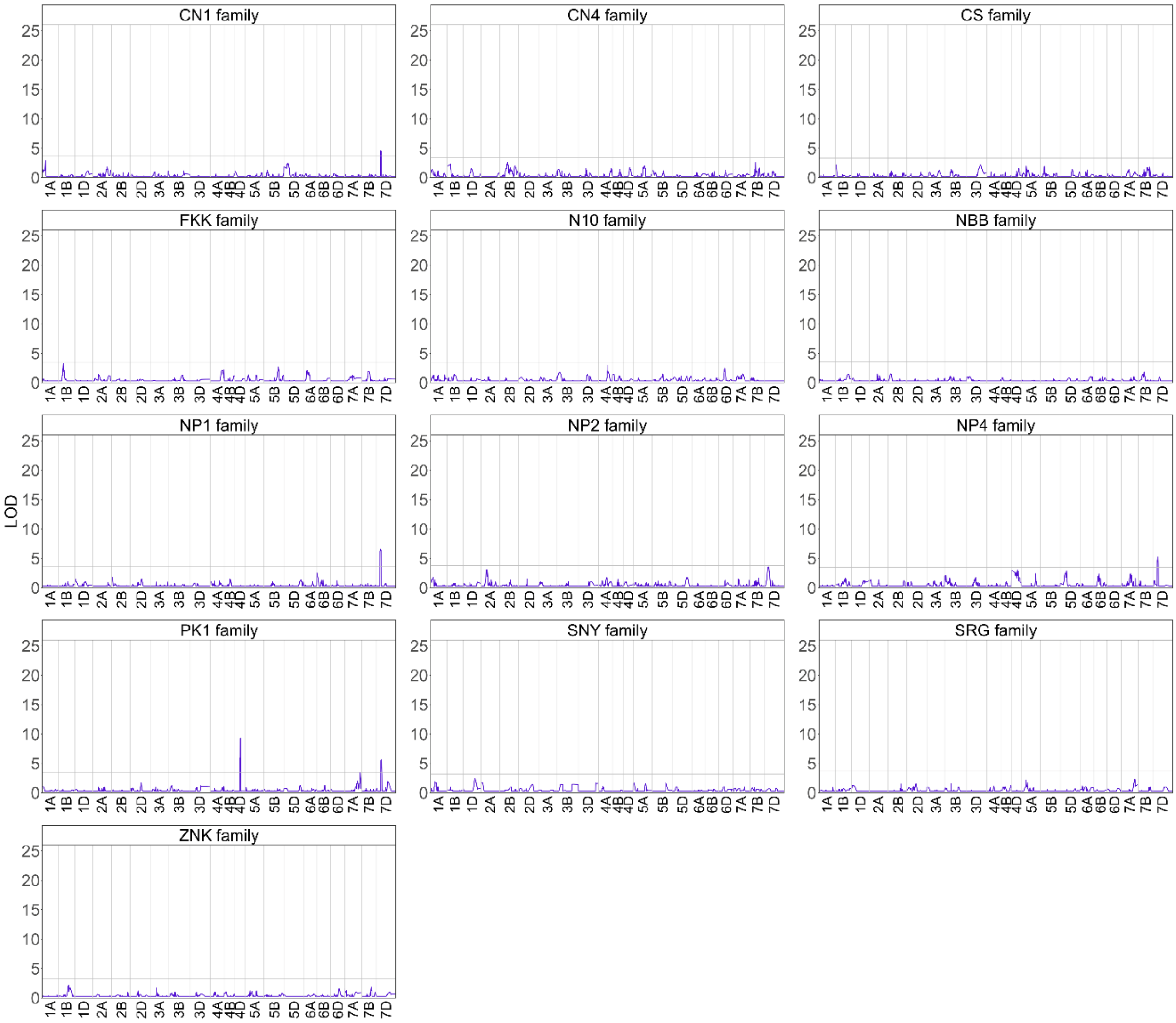

Supplementary Figure 18: Single-family QTL mapping for leaf rust data collected in Obregon 22.

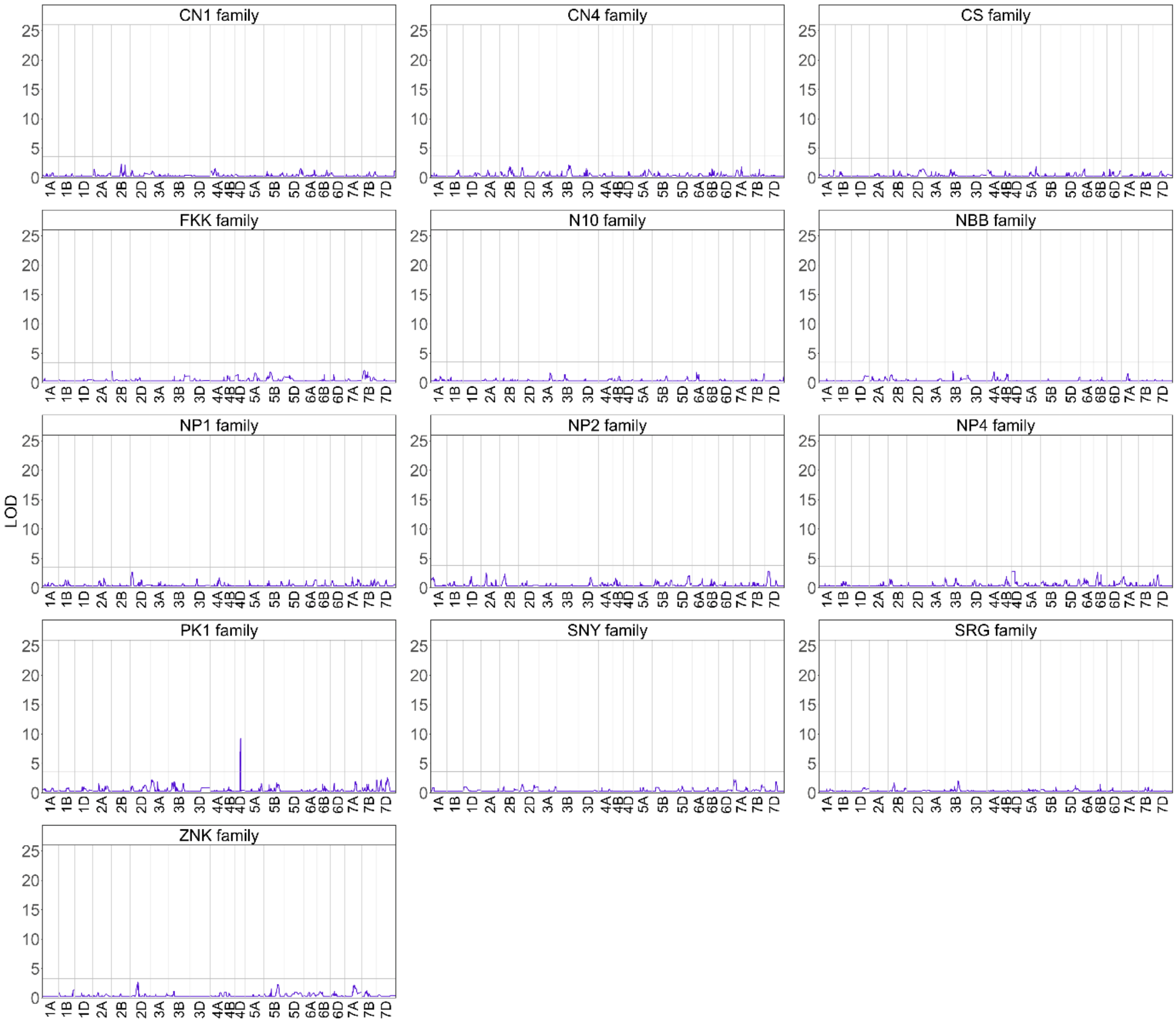

Supplementary Figure 19: Single-family QTL mapping for stem rust data collected in Obregon 22.

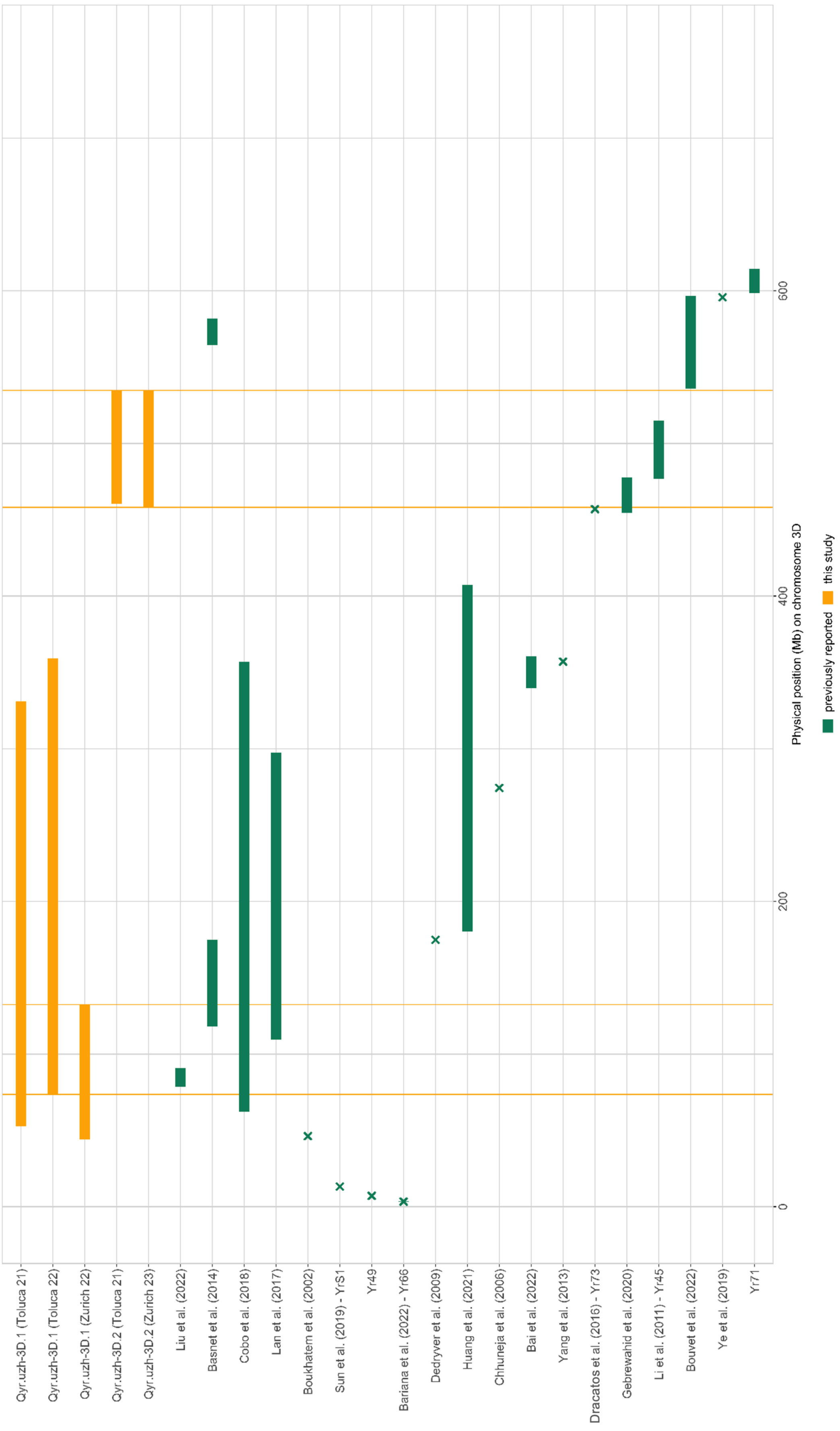

Supplementary Figure 20: Physical position of previously published QTLs and genes on chromosome 3D; detailed information is stored in Supplementary Table 15.

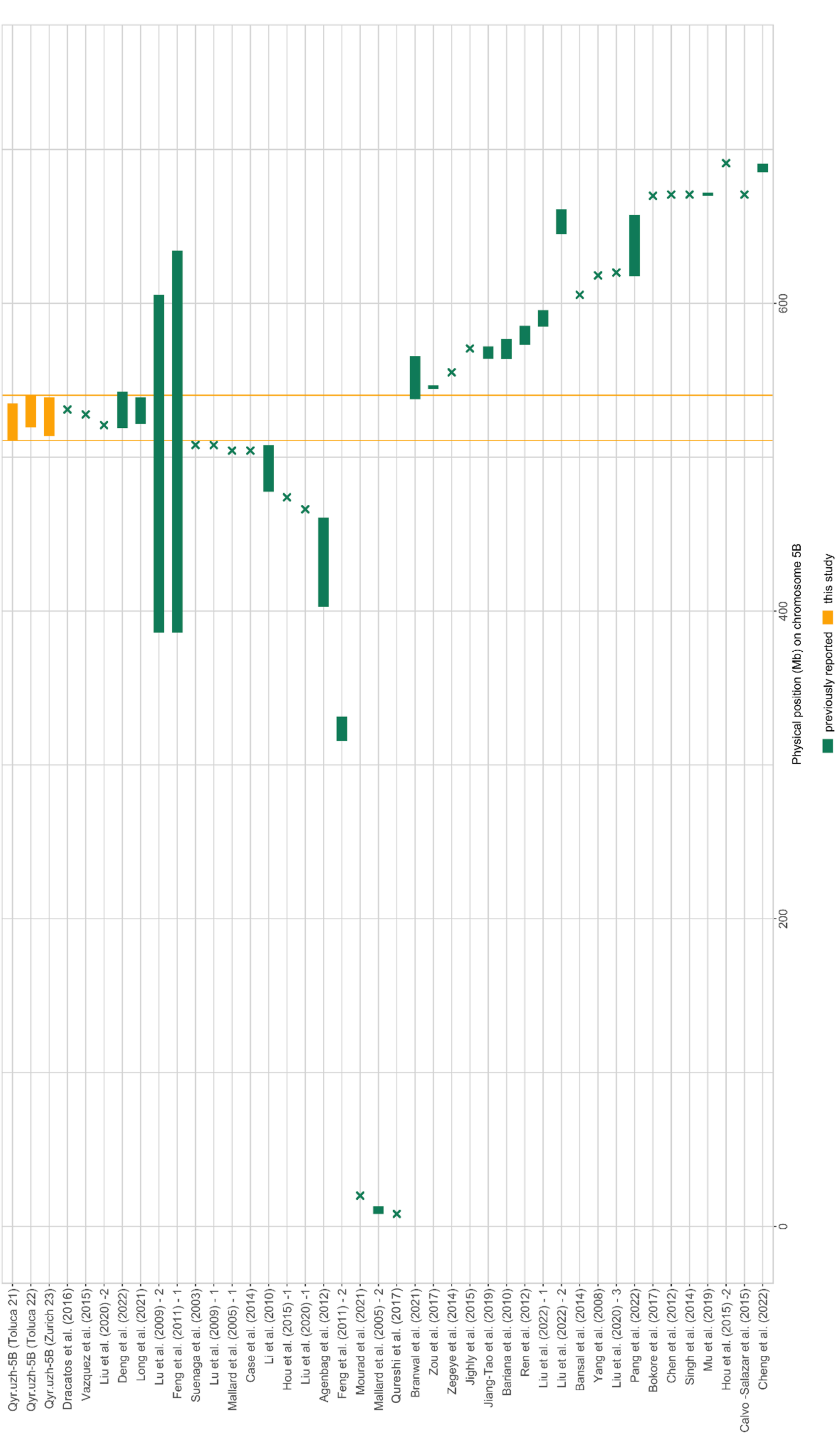

Supplementary Figure 21: Physical position of previously published QTLs and genes on chromosome 5B; detailed information is stored in Supplementary Table 16.
